# Behavioral Validation of Individualized Low-Intensity Transcranial Electrical Stimulation (tES) Protocols

**DOI:** 10.1523/ENEURO.0374-22.2023

**Published:** 2023-12-05

**Authors:** Rajat Joshi, Sainath Murali, Nivethida Thirugnanasambandam

**Affiliations:** 1 National Brain Research Centre (NBRC), Manesar 122 052, India; 2 Human Motor Neurophysiology and Neuromodulation Lab, Department of Biosciences and Bioengineering, Indian Institute of Technology Bombay, Powai Mumbai 400076, India

**Keywords:** implicit motor learning, individualized dose, tACS, tDCS, transcranial electrical stimulation (tES), working memory

## Abstract

Large interindividual variability in the effects of low-intensity transcranial electrical stimulation (tES) considerably limits its potential for clinical applications. It has been recently proposed that individualizing stimulation dose by accounting for interindividual anatomic differences would reduce the variability in electric fields (E-fields) over the targeted cortical site and therefore produce more consistent behavioral outcomes. However, improvement in behavioral outcomes following individualized dose tES has never been compared with that of conventional fixed dose tES. In this study, we aimed to empirically evaluate the effect of individualized dose tES on behavior and further compare it with the effects of sham and fixed dose stimulations. We conducted a single-blinded, sham-controlled, repeated-measures study to examine the impact of transcranial direct current stimulation on motor learning and that of transcranial alternating current stimulation on the working memory of 42 healthy adult individuals. Each participant underwent three sessions of tES, receiving fixed dose, individualized dose, or sham stimulation over the targeted brain region for the entire behavioral task. Our results showed that the individualized dose reduced the variability in E-fields at the targeted cortical surfaces. However, there was no significant effect of tES on behavioral outcomes. We argue that although the stimulation dose and E-field intensity at the targeted cortical site are linearly correlated, the effect of E-fields on behavior seems to be more complex. Effective optimization of tES protocols warrants further research considering both neuroanatomical and functional aspects of behavior.

## Significance Statement

Transcranial electrical stimulation (tES) has shown remarkable results in motor, cognitive, and clinical investigations. However, there is high variability in the efficacy of tES results with a small effect size and reproducibility. The E-field intensity variability at the targeted cortical site has been shown to contribute to a significant proportion of the variability in behavioral effects of tES. The variability in E-field intensity has recently been demonstrated to decrease with individualizing tES dosages. Consequently, it is widely assumed that uniform E-field intensity across individuals will lead to reduced variability in behavioral outcomes. In this study, we investigated the effect of individualized tES on motor and working memory behavior. Our results showed contradictory outcomes where individualized tES did not reduce the variability in behavioral outcomes.

## Introduction

Low-intensity transcranial electric stimulation (tES) is an emerging technique that modulates brain activity noninvasively and painlessly in alert individuals (Nitsche and Paulus, 2000; Nitsche et al., 2003, 2005; Boros et al., 2008; Fritsch et al., 2010). Typically, a direct or alternating current is used in tES investigations, thus called transcranial direct current stimulation (tDCS) and transcranial alternating current stimulation (tACS). tES is known to enhance motor (Nitsche et al., 2003, 2004; Antal et al., 2007; Boros et al., 2008; Fritsch et al., 2010), behavioral (Wach et al., 2013; Hannah et al., 2019), and cognitive (Kuo and Nitsche, 2012; Fröhlich et al., 2015; Talsma et al., 2017; Green et al., 2020) performance.

tES holds immense potential in the treatment of neuropsychiatric illnesses (Uhlhaas et al., 2009; Kuo et al., 2017; Sreeraj et al., 2017; Elyamany et al., 2021). However, tES efficacy shows mixed results across studies with small effect sizes and poor reproducibility, limiting the use of tES in clinical applications. A meta-analysis on schizophrenia showed no direct effects of tDCS on positive and negative effects, with only a subgroup of patients showing improvement in symptoms with multiple tDCS interventions (Ciullo et al., 2021). Additionally, various behavioral studies on motor learning have shown poor reproducibility or nonreproducibility of tES interventions (Kang and Paik, 2011; Stagg et al., 2011; Ambrus et al., 2016; Ammann et al., 2016). A meta-analysis study by Senkowski et al. (2022) showed mixed effects of tDCS and tACS on working memory behavior, with single-session tDCS having no effect and multisession tDCS and tACS having moderate effects.

The large variability in the effects of tES has been attributed to the interindividual anatomic differences (Kim et al., 2014; Laakso et al., 2015) and different stimulation parameters (Li et al., 2015) used across studies. Integrated current flow modeling and neuroimaging indicate that a large proportion of this variability is because of differences in the direction of current flow (Rawji et al., 2018) and the distribution of electrics fields (E-fields) generated in the brain (Kasten et al., 2019; Evans et al., 2020). tES studies typically use a fixed dose of 1–2 mA to stimulate the subjects’ brains without accounting for the interindividual anatomic differences. As a result, there is large variability in the E-fields generated on the cortical surface across individuals (Caulfield et al., 2022).

A recent study by Evans et al. (2020) showed a 100% reduction in variability in E-field intensity at the targeted cortical site by the dose individualization method. The proposed pipeline used current flow models to calculate the tDCS dose required to produce uniform E-fields across subjects. Assuming a direct linear relationship between the E-fields and physiological and behavioral effects, this approach was expected to reduce the variability in behavioral outcomes across individuals (Evans et al., 2020; Caulfield et al., 2022). However, this hypothesis has not been empirically validated.

In this single-blinded study, we empirically evaluated the effect of individualized tES dose on behavior. Further, we compared it with the effects of sham and fixed dose stimulation in healthy human adults. We targeted two brain regions: the left primary motor cortex to evaluate the effects of individualized tDCS on motor learning (Nitsche et al., 2003), and the right frontoparietal cortices to evaluate tACS on the working memory task (Violante et al., 2017), respectively. To achieve this, we adopted experimental parameters like behavioral task, electrode montage, and stimulation intensity from previous studies conducted by Nitsche et al. (2003) and Violante et al. (2017). We aimed to replicate the improvement in motor learning and working memory performance of individuals using tDCS and tACS, as shown by Nitsche et al. (2003) and Violante et al. (2017), respectively. We further hypothesized that individualized dose tES would produce a larger effect on the behavior because of less interindividual variability.

## Materials and Methods

### Study overview

**Figure 1.**
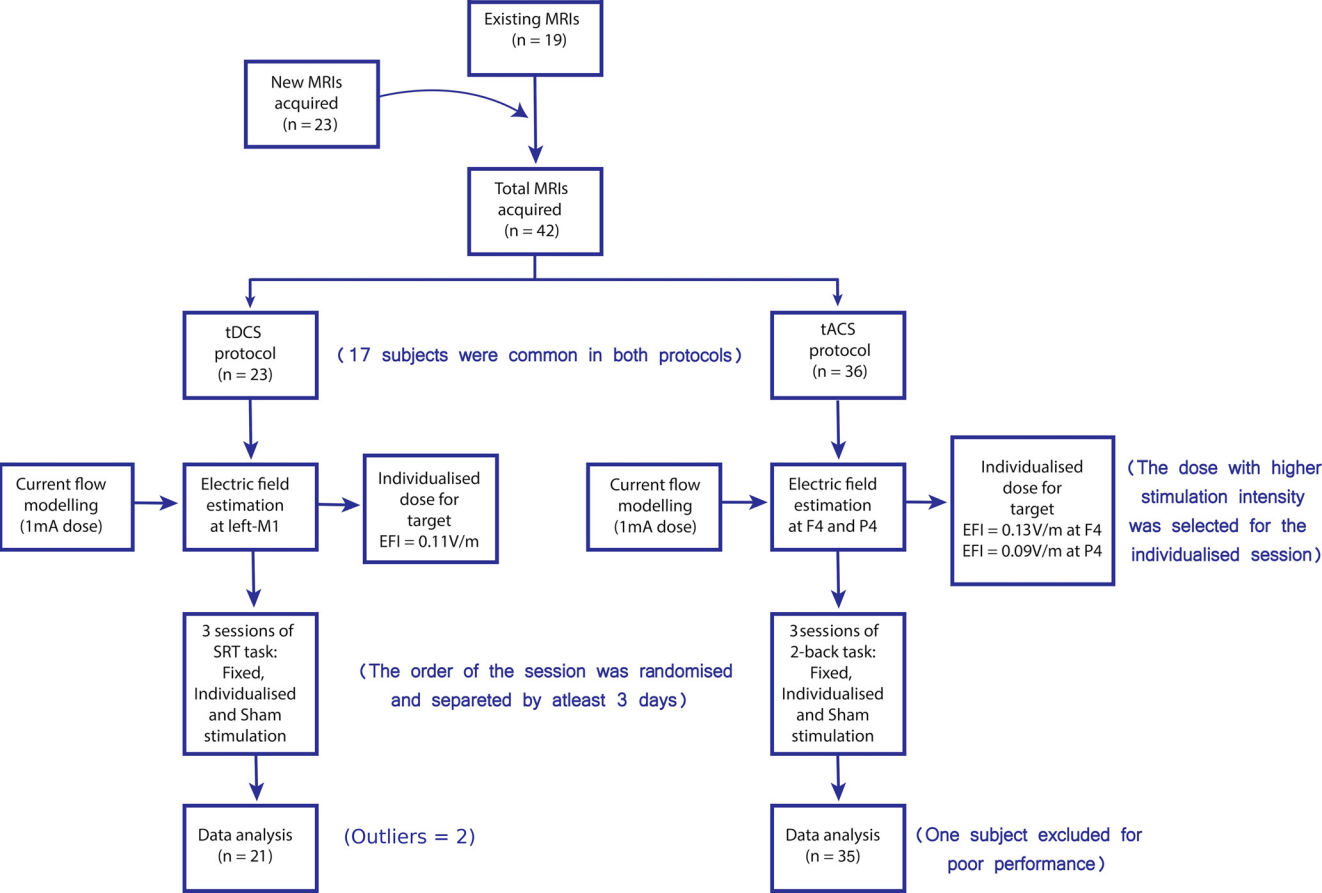
Study timeline.

Forty-two healthy adult volunteers were recruited for the study. The sample size was determined by statistical power analysis [Table 1] on the results of Nitsche et al. (2003) and Violante et al. (2017) for the tDCS and tACS experiments, respectively. The tDCS experiment had 23 participants (mean age = 26.00 ± 2.62 years; 16 females), while the tACS study had 36 participants (mean age = 25.14 ± 2.30 years; 25 females; Fig. 1). Seventeen subjects participated in both experiments. All participants were right handed (mean = 78.45 ± 24.95%) as evaluated by the Edinburgh handedness inventory (Oldfield, 1971). They all had a university or higher-level education, normal or corrected-to-normal vision, and no contraindications for tES (Kessler et al., 2012). Human subjects were recruited from the National Brain Research Centre (Manesar, India). The study conformed to the guidelines of the Declaration of Helsinki and was approved by the Institutional Human Ethics Committee of the National Brain Research Centre (NBRC; Manesar, India). All the participants signed a written informed consent before the commencement of the study.

**Table 1:** Power analysis table

	tDCS	tACS
Test	Paired, two-tailed *t* test	Paired, two-tailed *t* test
Difference in means	0.055	0.075
SD	0.09	0.15
Power	80%	80%
Type I error	5%	5%
Sample size	23	34

The sample size required for the tDCS and tACS experiments is 23 and 36, respectively. For both analyses, we used an a priori, paired, two-tailed *t* test to compute required sample sizes using G*Power version 3.1.9.6 (http://www.gpower.hhu.de/). For the tDCS experiment, the expected effect size was chosen as the mean reaction time difference (ms) between the last learning block (block 5) for the sham and the anodal tDCS condition from the Nitsche et al. (2003) paper. Similarly, the expected effect size of the tACS experiment was the mean time difference (ms) between the sham and synchronous tACS conditions for the 2-back task at 6 Hz from Violante et al. (2017). Thus, based on the above studies, we expected a minimum difference in the means to be 55 and 75 ms between the conventional and individualized dose conditions for tDCS and tACS experiments.

10.1523/ENEURO.0374-22.2023.f1-1 Figure 1-1 ***A***, The SRTT. Each trial displayed four outlined boxes and a target, which is a solid red square. The target changed its position every trial over each box. The subjects were asked to place their right-hand index, middle, ring, and little finger over the keys labeled 1, 2, 3, and 4, respectively. They registered their response by pressing the key corresponding to the target position and the next target appeared 500 ms later. The task consisted of 8 blocks with 120 trials each. The target was presented in a pseudorandom fashion in blocks 1 and 6 in equal frequency over each position, without appearing consecutively at the same position. The rest of the blocks followed the same sequence of 12 trials (e.g., 341212342341) repeated 10 times. Participants performed 20 practice trials at the start of the first session, with visual feedback provided at the end of each trial. Subjects were unaware of the repeating sequence, and a unique sequence was used for every participant for every session. ***B***, The 2-back task. A sequence of numbers, ranging from 0 to 9, was presented one at a time in a randomized order. The subjects were asked to register the response using their right index finger when the currently displayed number matched the one presented two trials earlier by pressing the left mouse button. The sequence consisted of 200 trials with 50 targets and 150 distractors (nontarget number). Download Figure 1-1, TIF file.

### Structural MRIs

Structural T1-weighted MR images were obtained for each participant. We had access to the MRI scans of 19 participants in the NBRC database in neuroimaging informatics technology initiative (NIFTI) format. The data were obtained from an Achieva 3.0 T MRI scanner (Philips) with the following parameters: TR = 8.39 ms; TE = shortest; FOV = 250 × 230.16 × 170 mm; flip angle = 8°; voxel size = 1 mm^3^; scan duration = 5 min, 28.5 s. The other 23 scans were acquired in a 3 T MAGNETOM Prisma MRI scanner (Siemens). Data were obtained using the following protocol: TR = 2300 ms; TE = 2.33 ms; FOV = 240 mm; voxel size = 0.9 mm^3^; flip angle = 8°; scan duration = 5 min, 21 s. The raw MR images were saved in the digital imaging and communications in medicine (DICOM) format and converted through MRIcroGL 1.2.20211006 × 86–64 FPC to NIFTI format.

### Current flow modeling

We used Realistic Volumetric Approach to Simulate Transcranial Electric Stimulation (ROAST) and SPM12 for current flow modeling and E-fields extraction (Huang et al., 2013, 2019). Further, we adapted the pipeline devised by Evans et al. (2020; https://github.com/caryse/tDCS_dosecontrol) to compute the individualized dose and extract E-field intensities from the target site (Evans et al., 2020). The method uses current flow models coupled with neuroimaging to calculate the tES intensities. Individualization accounted for the interindividual differences in anatomic characteristics and delivered a uniform current to the targeted cortical site across individuals.

#### Regions of interest

**Figure 2.**
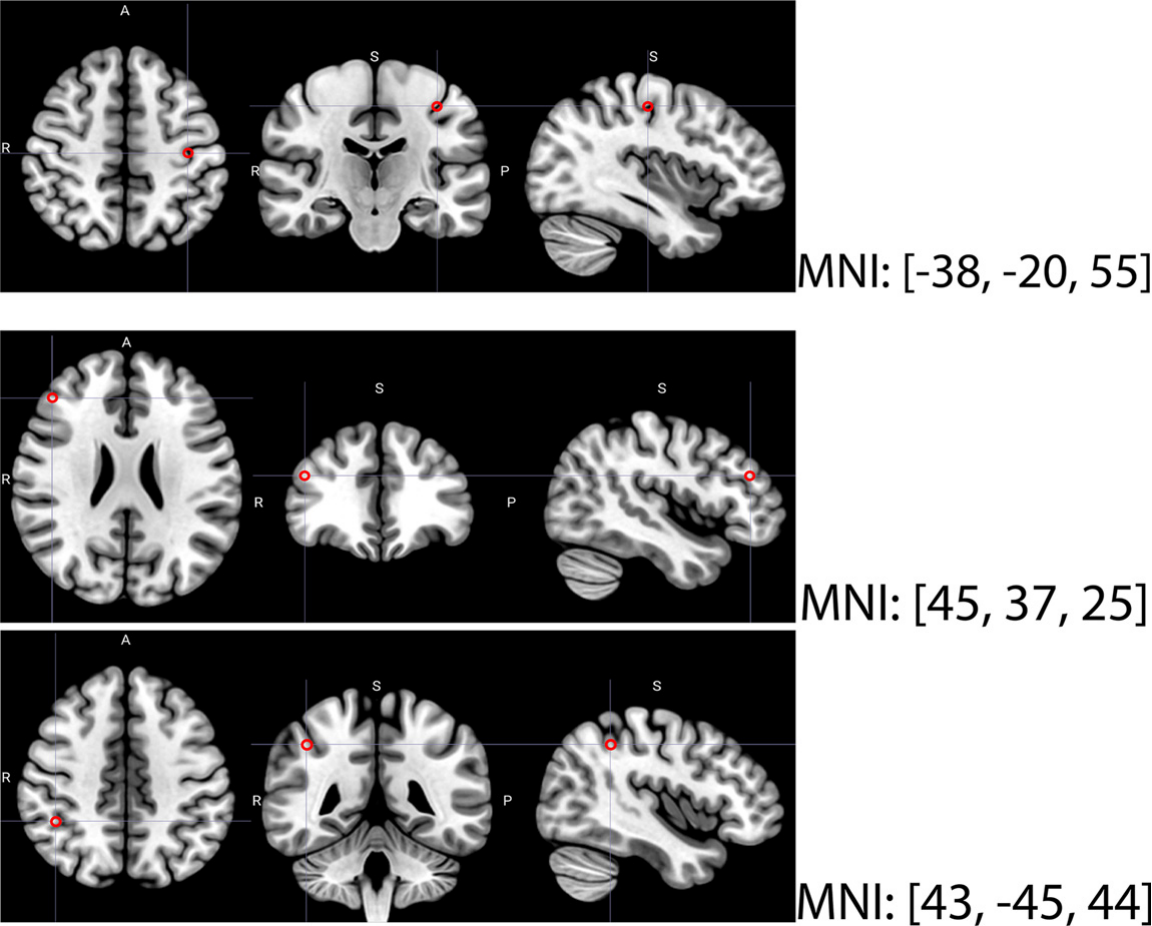
The ROIs. We targeted the left primary motor cortex, the left M1 (MNI: −38, −20, 55), for the tDCS experiment (top). For the tACS experiment, we targeted the right middle frontal gyrus (MNI: 45, 37, 25; middle) and right inferior parietal lobule (MNI: 43, −45, 44; bottom). The center of the red ring marks the targeted coordinates in radial, axial, and sagittal axes.

To access the E-field intensity at the target regions—left primary motor cortex (M1; for the tDCS experiment) and frontoparietal cortices (for the tACS experiment)—eigen variates were extracted from spherical regions of interest (ROIs) with a 5 mm radius using SPM12. The MNI coordinates for M1 (MNI: −38, −20, 50) were based on the activation likelihood estimation results by Eickhoff et al. (2009). Similarly, we used ROIs in the right middle frontal gyrus (MNI: 45, 37, 25) and right inferior parietal lobule (MNI: 43, −45, 44) under F4 and P4 electrodes, respectively (Fig. 2). The coordinates for these regions were taken from a meta-analysis study by Wang et al. (2019). These coordinates were in Talairach format and thus required conversion to MNI, which was done by a transformation matrix (https://www.brainmap.org/icbm2tal/tal2icbm_spm.m).

#### Electric field extraction

To extract the E-field intensities from the target regions, we adhered to the pipeline suggested by Evans et al. (2020). The ROAST structural MR and E-field images were processed using SPM12 with the following pipeline: structural and E-field images were resampled to 2 × 2 × 2 mm voxels and smoothed using a 4 mm full-width at half-maximum Gaussian kernel. Following that, a binary mask of gray and white matter was created using the normalized-smoothed structural images to restrict voxel analysis inside the brain. The mask was created by averaging the individual nonbinary gray and white matter tissue masks generated by ROAST, with an inclusion threshold of >0.1.

### Dose individualization

The doses were individualized for a target E-field intensity calculated by averaging the mean of the E-fields generated in the target brain regions across all the participants with a stimulation intensity of 1 mA. The stimulation intensity of 1 mA was used to limit the spatial distribution of the E-fields in nontarget regions. We obtained three target E-field intensities for the three targets. The target E-field intensity for left M1 was 0.1135 ± 0.021 V/m; for the right middle frontal gyrus and inferior parietal lobule, the target E-field intensities were 0.1267 ± 0.034 and 0.0968 ± 0.021 V/m (all the values rounded off to fourth decimal), respectively (Evans et al., 2020; Caulfield et al., 2022). The doses were then individualized using the following equation:

$$\frac{\text{Individualized dose}}{\text{ Group average E-field}}\text{ = }\frac{\text{1 mA Dose}}{\text{E-field from 1 mA}}.$$

Rearranging the equation, we get the following:

$$\text{Individualized dose = }\frac{\text{1 mA Dose × Group average E-field }}{\text{E-field from 1 mA}}.$$

For the tDCS experiment, individualizing stimulation doses was effective in generating uniform E-fields over the left M1 across the participants (i.e., stimulation was both individualized and dose controlled). In the tACS experiment, although we individualized the stimulation doses since there were two active electrodes, we could deliver dose-controlled stimulation only to one of the two sites. This is because the tES device we used could not deliver unequal intensities of current over two sites simultaneously. Hence, the stimulation dose was determined by the dose individualized over one of the electrodes. Thus, for the tACS experiment, although the stimulation was individualized, it was not completely dose controlled. Nevertheless, we expected that individualizing the dose over one of the electrodes would affect the E-field over the other site in a similar manner and therefore reduce variability.

### tES

tES was delivered using Nurostym tES (Brainbox). The participants were blinded to the stimulation and underwent three sessions where they received a fixed dose of 1 mA, an individualized dose, or sham tES. The sessions were randomized across participants and scheduled at least 3 d apart. We calculated the individualized dose for tES using the reverse calculation approach described above. Sham stimulation involved a brief stimulation lasting 30 s with a 20 s ramp-up period, giving the impression of active stimulation without exerting any physiologically significant effects (Kessler et al., 2012).

#### tDCS

**Figure 3.**
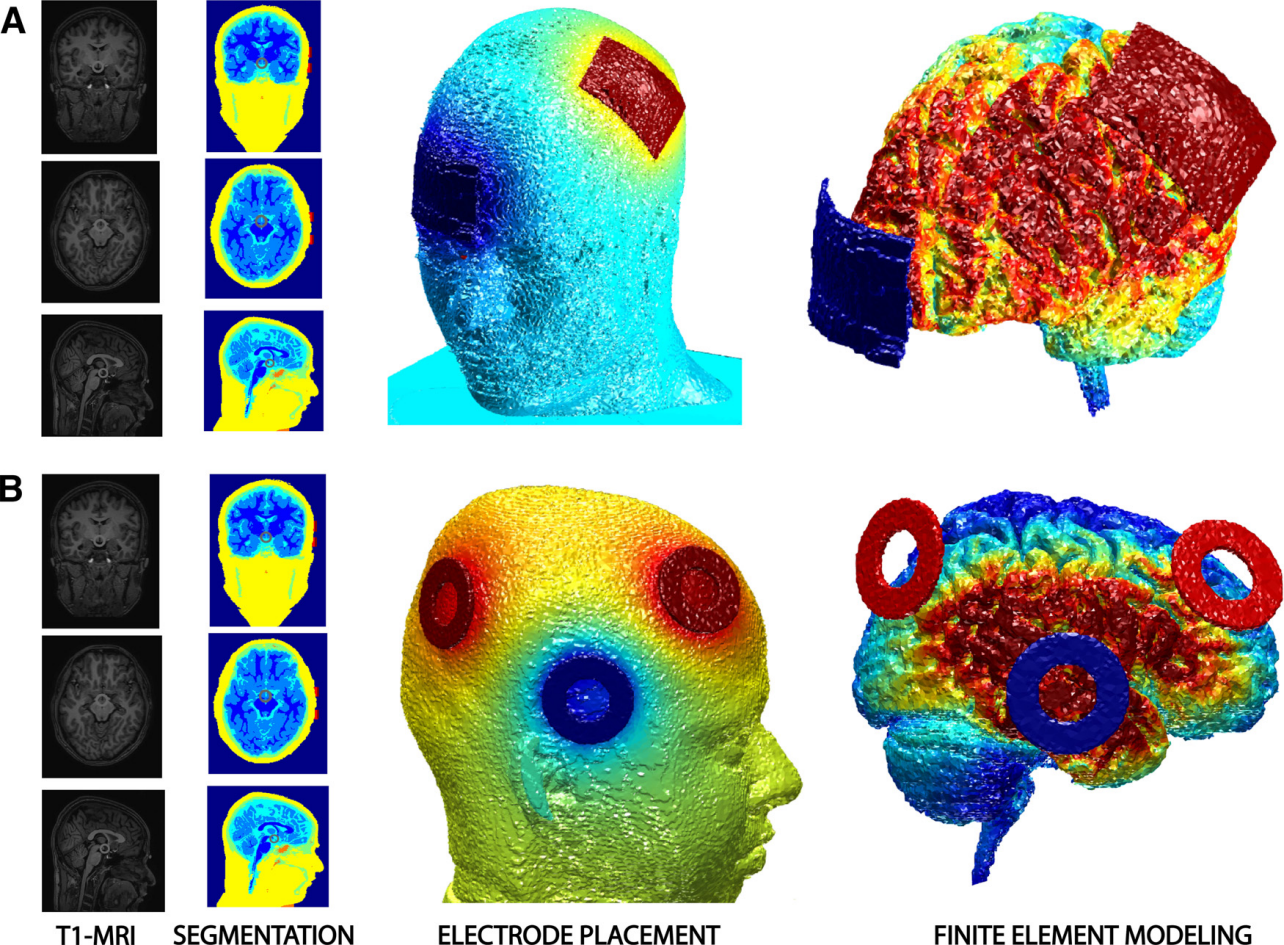
The figure shows the ROAST processing steps. In the first step, the MRI undergoes the process of tissue segmentation. The subsequent steps include electrode placement, meshing, and finite element modeling. ***A***, Electrode montage for the tDCS experiment. The anode (red) is positioned over 10-10 EEG coordinates C3 (Left M1), and the return (blue) is placed over the contralateral supraorbital region (Fp2). ***B***, Electrode montage for the tACS experiment. The anodes (red) are positioned over the 10-10 EEG coordinates F4 (middle frontal gyrus) and P4 (Inferior parietal lobule), whereas the return is placed over T8 (temporal cortex).

Saline-soaked pad type (7 × 5 cm; area = 35 cm^2^) sponge electrodes were used for tDCS stimulation. The anode was placed over the C3 (left M1), and the cathode was placed over the Fp2 (contralateral forehead; Nitsche et al., 2003; Fig. 3*A*). The longer axis of the electrode was placed along the mediolateral direction. The impedance was kept <20 kΩ during the stimulation with the electrodes held in position using rubber headbands. The stimulation lasted for the entire session (through blocks 1–8), ∼17 ± 1.95 min. For the fixed dose session, subjects received continuous stimulation of 1 mA with a 20 s ramp-up period at the start of the experiment. Individualized dose sessions had a unique current dose with a 20 s ramp-up period for each participant. In contrast, in sham sessions, participants received stimulation for 30 s at the start of the experiment, which did not affect performance.

#### tACS

Three ring-shaped (inner radius = 12 mm; outer radius = 24 mm) rubber electrodes were used for the experiment. Anode electrodes were placed on the 10-10 coordinates F4 (middle frontal gyrus) and P4 (inferior parietal lobule), and the cathode was placed over the T8 (temporal cortex; Fig. 3*B*). Each participant underwent three sessions of tACS stimulation with a stimulation frequency of 6.00 Hz and without DC offset (Violante et al., 2017). To keep the impedance <20 kΩ and to maintain the electrodes in place, we used Ten20 (Weaver) conductive paste. The participants wore a mesh cap to further prevent electrodes from falling. Finally, the whole setup was held together using a rubber headband. For the fixed dose sessions, participants received a peak-to-peak amplitude of 1 mA with a 20 s ramp-up period at the start of the experiment.

The current amplitude varied in the individualized sessions. Two target intensities were obtained for calculating the individualized dose, one for each anode over 10-10 coordinates F4 and P4. We selected the higher of the two individualized doses for stimulation to ensure strong enough E-fields over both sites. Thus, it is important to note that although the tACS intensity was individualized, it was dose controlled only over one of the two sites. The stimulation lasted for the entire session, which was ∼7.85 ± 0.83 min. In sham sessions, participants received stimulation only for the initial 30 s of the experiment with 1 mA peak-to-peak amplitude.

### Behavioral tasks

The participants performed the serial reaction time task (SRTT) that assesses implicit motor learning for the tDCS experiment (as described by Nitsche et al., 2003) and the 2-back task for the tACS experiment that assesses working memory (as described by Violante et al., 2017).

In the SRTT, the participants performed finger movements repeatedly without being aware of a sequential order. The task consisted of eight blocks; blocks 2–5 and 7–8 (learning blocks) had the same sequence to be performed repeatedly. In blocks 1 and 6 (random blocks), the sequence was random (Extended Data Fig. 1-1*A*). The difference between learning block 5 and random block 6 was taken as a measure of implicit learning.

The 2-back task consisted of a single block of 200 trials, with 50 target and 150 nontarget trials. The subjects were asked to register the response using their right index finger when the currently displayed number matched the one presented two trials earlier by pressing the left mouse button (Extended Data Fig. 1-1*B*).

### Data analysis

#### Serial reaction time task

We used the Nitsche et al. (2003) pipeline for the SRTT behavioral analysis (Nitsche et al., 2003). The reaction time (RT) was measured as the time from the presentation of the target to the time of response. The subject-wise analysis consisted of only the correctly responded trials; the incorrect trials and correct trials with RTs >3000 or <200 ms and trials with RT >3 SDs of the individual’s mean RT were discarded. A total of 933.3 ± 17.53 of 960 trials was left for analysis. The mean RTs were normalized to that of the first block. The *z* scores showed two participants above and below the 3 SDs of the mean. Hence, they were excluded from further analysis. The normality test (Shapiro–Wilk test) revealed a non-normal distribution of data. Thus, we opted for a nonparametric two-way repeated-measures ANOVA for further analysis. The nonparametric ANOVA was performed using the ARTool package (Wobbrock et al., 2011), which implements the Aligned Rank Transform (ART) to conduct multifactorial nonparametric ANOVA while accounting for the repeated measures. The independent variables were block (eight levels) and stimulation condition (three levels; fixed, individualized, and sham). All blocks were normalized to block 1, which was considered as the individual baseline. Block 1 was included in the ANOVA so as to estimate the participants' performance relative to the baseline. *Post hoc* analysis was done using the ART contrast to perform multiple comparisons with Bonferroni adjustments (Elkin et al., 2021).

#### 2-back task

The primary behavioral outcomes for this task were RT, accuracy, and d′ (a measure of sensitivity). The RT was measured as the time difference between the target onset and response. The accuracy was estimated as follows:

$$\text{Accuracy = }\frac{\text{TP + TN}}{\text{TP + TN + FP + FN}},$$

where TP is true positives, TN is true negatives, FP is false positives, and FN is false negatives. The accuracy was then converted to a percentage. In addition to RT and accuracy, we calculated d′, which takes into account the false alarm rate along with the hits to correct the response bias. The d′ was computed using the following formula:

$$d^{\text{'}}=Z\left(\text{Hit rate}\right)\text{ - }Z\left(\text{False alarm rate}\right).$$

The *Z* denotes *Z*-transform. The hit rate and false alarm rate were calculated as follows:

$$\text{Hit rate = }\frac{\text{Hit}}{\text{Hit + Miss}},$$

$$\text{False alarm rate = }\frac{\text{False alarm}}{\text{False alarm + Correct rejections}}.$$

Next, to examine the effect of E-field intensity (V/m) and stimulation dose (mA) on performance, we computed Pearson’s correlation between the RTs and the E-field intensity, and the RT and Stimulation dose. The E-field intensity was extracted from regions under 10-10 EEG coordinates F4 and P4.

## Results

### Current flow modeling

**Figure 4.**
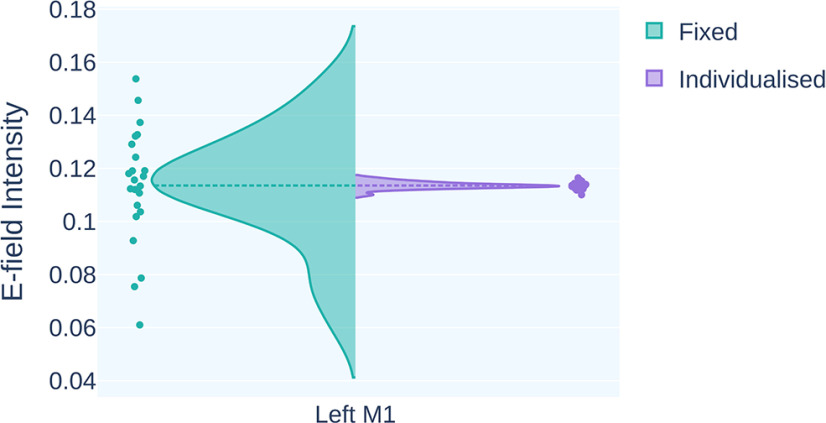
The figure shows the distribution of E-field intensity in left M1 (MNI: −38, −20, 50) for fixed (green) and individualized doses (purple). The variance in E-field intensity was reduced by 99.86% after individualizing the dose. The individual E-field intensity for 1 mA is normalized to the mean E-field (0.114 V/m) following dosage individualization. Extended Data Figure 4-1, *A* and *B*, shows the quantitative comparison of the E-fields in the brain for fixed and individualized doses.

For tDCS, the mean E-field generated over left M1 (MNI: −38, −20, 50) was 0.114 ± 0.023 V/m (range, 0.061–0.154 V/m) for 1 mA. This value was used as the target E-field intensity for calculating the individualized stimulation dose (Dose = 1.045 ± 0.30 mA; range = 0.738–1.860 mA). The variance in E-field intensity was reduced by 99.86% after dose individualization. Dose individualization normalized the individual E-field intensity to the target E-field intensity of 0.114 V/m (Fig. 4). Extended Data Fig. 4-1*A* describes the qualitative comparison of E-field intensity at left M1.

10.1523/ENEURO.0374-22.2023.f4-1 Figure 4-1 ***A***, ***B***, The figures show the qualitative comparison of the electric field generated over the left-M1 (***A***) and frontoparietal cortex (***B***). For the subjects with low electric field intensity in the fixed dose condition, the individualized dose increased the electric field to approximately the mean electric field. In contrast, the electric field was decreased for the subjects with high electric field generation in the fixed dose condition. Download Figure 4-1, TIF file.

10.1523/ENEURO.0374-22.2023.f4-2 Figure 4-2 The stimulator output (mA) distribution for electric field intensity (V/m) in left-M1 and frontoparietal cortex. Download Figure 4-2, TIF file.

**Figure 5.**
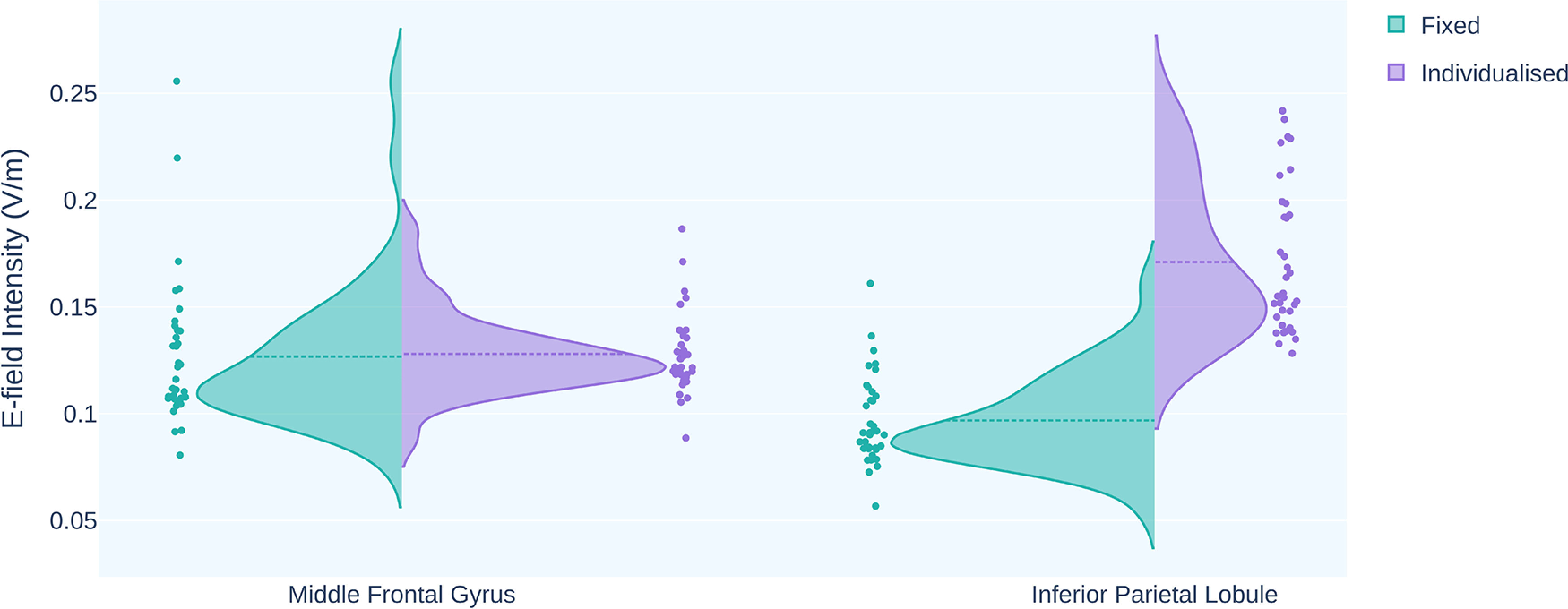
The E-field intensity distribution at frontoparietal cortices for fixed (green) and individualized (purple) doses. The variance in the distribution of E-field intensity at middle frontal gyrus (MNI: 45, 37, 25) was reduced by 69.92% after dose individualization. The selection of a higher individualized dose for targets F4 and P4 increased the variance at the inferior parietal lobule (MNI:43, −45, 44) by 168.59%. The higher individualized dose simultaneously stimulated both the targeted regions.

tACS applied to the frontoparietal cortices at a fixed dose of 1 mA (peak-to-peak amplitude) generated varying E-field intensities at the middle frontal gyrus (MNI: 45, 37, 25) and inferior parietal lobule (MNI: 43, −45, 44). The mean field intensities were 0.127 ± 0.034 V/m (range = 0.080–0.256 V/m) and 0.097 ± 0.021 V/m (range = 0.057–0.161 V/m) at the middle frontal gyrus and inferior parietal lobule, respectively, resulting in two different individualized doses. We used the higher of the two doses to stimulate both regions (Dose = 1.219 ± 0.193 mA; range = 0.893–1.748 mA). This caused the variance in E-field intensity (V/m) to reduce by 69.92% at the middle frontal gyrus, while it increased by 168.59% at the inferior parietal lobule (Fig. 5). Most importantly, this ensured that the E-field intensity over the stimulation sites was either equal to or greater than that generated by a fixed 1 mA current (Extended Data Fig. 4-1*B* describes the qualitative comparison of E-field intensity at frontoparietal cortices). To create homogeneous E-field intensity at the targeted cortical regions, the variability in stimulator output increased because of interindividual anatomic variances (Extended Data Fig. 4-2).

### Behavioral data analysis

#### tDCS

**Figure 6.**
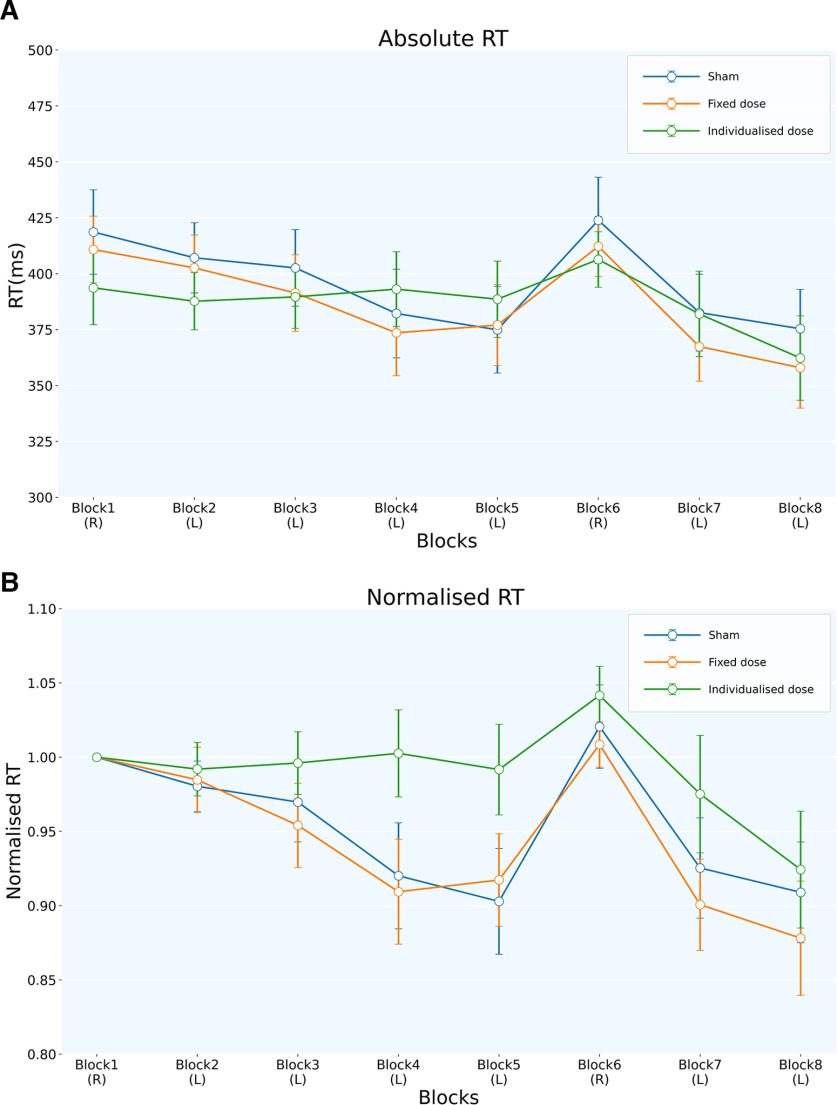
The figure shows the performance across blocks for all the tDCS conditions. The *y*-axis represents absolute RT (ms) for ***A***, whereas the RT (ms) is normalized to block 1 in ***B***. R, Random block; L, learning block. ***A***, The figure shows reduced RT for the stimulation conditions as compared with the sham condition. However, there is no significant difference between conditions as tested by ANOVA. The sham and the fixed dose conditions show the learning curve across blocks 2–5. ***B***, The main effect of blocks and tDCS conditions was found, with individualized dose conditions significantly different from fixed dose and sham stimulation conditions. A trend of reducing RTs, reflecting implicit learning across learning blocks, is seen for the fixed dose and sham conditions. However, the individualized dose condition did not show this trend. Extended Data Figure 6-1 shows mean block-wise performance for all participants across tDCS sessions.

There was no significant difference in the absolute RT for block 1 (Q(2) = 2.667; *p* = 0.264; Kendall’s W = 0.063; Friedman test) across different stimulation conditions. The normalized RT across blocks showed a significant main effect of both blocks (*F*_(7,460)_ = 5.001; *p* < 0.001; η^2^ = 0.071; ANOVA by ART procedure) and condition (*F*_(2,460)_ = 8.381; *p* < 0.001; η^2^ = 0.035; ANOVA by ART procedure), but with no significant block × condition interaction (Fig. 6, Table 2). *Post hoc* analysis showed that the individualized dose condition differed significantly from fixed and sham conditions [Fixed::Individualized: *t*_(480)_ = −3.304; *p* = 0.003; Cohen’s *d* = −3.605; Individualized::Sham: *t*_(480)_ = 2.633; *p* = 0.02; Cohen’s *d* = 0.287; Tukey’s HSD; Extended Data Table 2-1]. The absolute (Fig. 6*A*) and normalized (Fig. 6*B*) RT data for sham and fixed dose conditions followed a trend of decreasing RTs through blocks 2–5, reflecting implicit motor learning for these conditions. Contrary to our expectation, we did not find this trend for the individualized dose condition.

**Table 2:** Results of 2-factorial repeated-measures ANOVA for the differential effects of blocks and tDCS conditions

Term	df	df.res	Mean squared	*F* value	*p* (>*F*)	η^2^ part	Significance
Block	7	460	74,029.1519	5.0009	0.00002	0.0707	***
Condition	2	460	123,664.6250	8.3805	0.0003	0.0352	***
Block:condition	14	460	14,894.9014	1.0052	0.4466	0.0297	

***p < 0.001. df, degree of freedom; df.res, degree of freedom residuals; η^2^, eta squared (partial).

10.1523/ENEURO.0374-22.2023.tab2-1 Table 2-1. (a) *Post hoc* analysis for the main effect of blocks. The Tukey’s HSD was used for comparisons, and the resulting *p*-values were corrected for multiple comparisons using Bonferroni correction. (b) *Post hoc* analysis for the main effect of conditions. The Tukey’s HSD was used for comparisons, and the resulting *p*-values were corrected for multiple comparisons using Bonferroni correction. Download Table 2-1, DOCX file.

10.1523/ENEURO.0374-22.2023.f6-1 Figure 6-1 The figure shows participants’ performances across tDCS sessions. The *y*-axis represents absolute RT (ms) and normalized RT in ***A*** and ***B***, respectively. R, Random block; L, learning block. ***A***, The absolute RT (ms) showed a significant main effect of blocks (*F*_(7,460)_ = 7.589; *p* < 0.001; η^2^ = 0.104), and sessions (*F*_(2,460)_ = 64.654; *p* < 0.001; η^2^ = 0.219), but no interaction. Participants’ performances at session 1 was significantly better than at the subsequent sessions. However, the performance in sessions 2 and 3 was not significantly different. ***B***, The normalized RTs showed a significant main effect of blocks (*F*_(7,460)_ = 7.204; *p* < 0.001; η^2^ = 0.099), sessions (*F*_(2,460)_  = 26.275; *p* < 0.001; η^2^ = 0.102), and also interaction (*F*_(14,460)_ = 1.7607; *p* = 0.042; η^2^ = 0.051). The results indicate a significant difference in participants’ performances between the first session and the subsequent sessions. However, there was no significant difference in performances between the second and third sessions. Download Figure 6-1, TIF file.

We also examined the impact of session number on performance as a control measure using two-way repeated-measures ANOVA. The ANOVA results showed a significant main effect of block (*F*_(7,460)_ = 7.204; *p* < 0.001; η^2^ = 0.099; ANOVA by ART procedure), session (*F*_(2,460)_ = 26.275; *p* < 0.001; η^2^ = 0.102; ANOVA by ART procedure), and block × session interaction (*F*_(14,460)_ = 1.7607; *p* = 0.042; η^2^ = 0.051; ANOVA by ART procedure) for the normalized RT (Extended Data Fig. 6-1*B*, Table 5). The *post hoc* analysis showed that participants performed significantly better in the first session than in subsequent sessions [Session1::Session2: *t*_(480)_ = −3.885; *p* < 0.001; Cohen’s *d* = −0.424; Session1::Session3: *t*_(480)_ = −3.790; *p* < 0.001; Cohen’s *d* = −0.414; Tukey’s HSD; Extended Data Table 5-1]. We did not find a significant difference between the performances in sessions 2 and 3 [Session2::Session3: *t*_(480)_ = 0.095; *p* = 0.995; Cohen’s *d* = 0.010; Tukey’s HSD; Extended Data Table 5-1].

**Figure 7.**
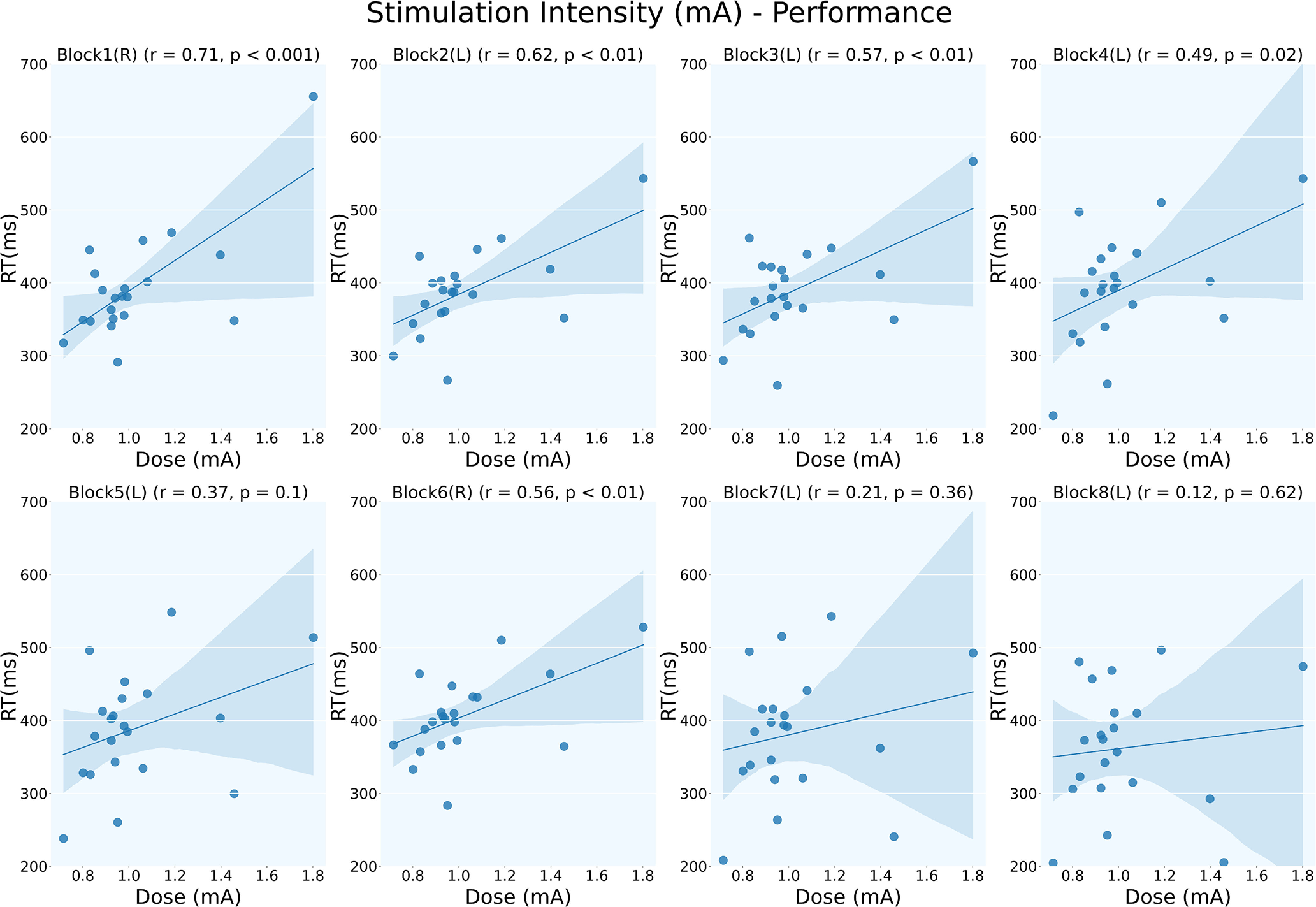
Block-wise correlation between stimulation dose and performance. Pearson’s *r* showed a positive correlation between stimulation dose and RTs. The randomized blocks (blocks 1 and 6, marked “R”) showed a strong positive correlation, whereas the correlation coefficient across the learning blocks (block 2 to block 4) shows a reducing trend. Learning blocks 5, 7, and 8 do not show a significant correlation. The trend in correlation shows the effect of learning and stimulation doses, with the higher dose of stimulation hampering learning. Extended Data Figure 7-1 shows block-wise correlation between E-field Intensity (V/m) and performance (ms).

Next, we computed Pearson's correlation between the stimulation dose and RT across the blocks. Block 1 showed the maximum positive correlation (*r*_(21)_ = 0.705; *p* < 0.001) between the individualized dose and RT, indicating that the participants who received a higher dose of stimulation performed poorly in the task. We also found a trend in the correlation coefficient values across the blocks. There was a gradual reduction in Pearson’s *r* from blocks 1–4, which became nonsignificant at block 5 (block 2: *r*_(21)_ = 0.619, *p* = 0.003; block 3: *r*_(21)_ = 0.568, *p* = 0.007; block 4: *r*_(21)_ = 0.488, *p* = 0.025; block 5: *r*_(21)_ = 0.371, *p* = 0.097). However, the correlation became positively significant in block 6 (*r*_(21)_ = 0.556; *p* = 0.009), the random block, and then again turned nonsignificant in the successive learning blocks (Fig. 7). This trend indicates the effect of stimulation dose on implicit learning of the sequence. The correlation between E-field intensity (V/m) and RT (ms) did not show a significant relationship for any of the blocks (Extended Data Fig. 7-1).

10.1523/ENEURO.0374-22.2023.f7-1 Figure 7-1 The graph shows the Pearson’s correlation between the electric field intensity (V/m) and the mean RTs (ms) for each block in the fixed dose condition for 21 subjects. R, Random block; L, learning block. The electric field intensity and the RT have a nonsignificant negative correlation (*p* > 0.05; Pearson's correlation) for all the blocks. The electric field intensity was computed from left M1 for a fixed dose of 1 mA tDCS stimulation. Download Figure 7-1, TIF file.

#### Differential effect of stimulation intensity on performance

**Figure 8.**
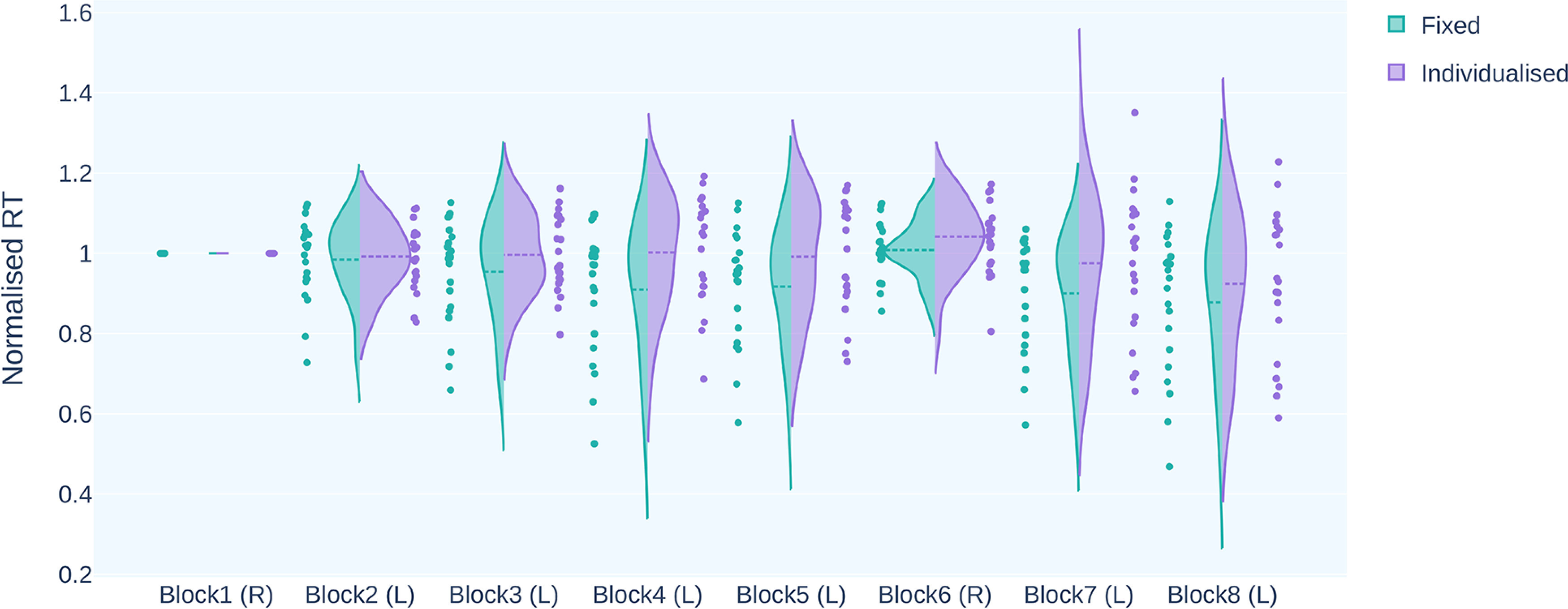
The plot shows the distribution of RT for every block across active tDCS conditions. R, Random block; L, learning block. The variability in performance is similar across both conditions. For the individualized dose condition, the learning blocks 2, 3, and 4 showed a bimodal distribution, indicating a differential effect of stimulation on performance. Extended Data Figure 8-1 shows the distribution of RT for the sham condition.

We did not find any difference in the variance in RT across the tDCS conditions. However, the performance in blocks 2–4 in the individualized dose condition showed a bimodal distribution, indicating a differential effect of stimulation on performance (Fig. 8, Extended Data Fig. 8-1). Based on this finding, we extended our analysis to explore the effect of stimulation intensity. For this, we divided the study subjects into the following two categories: those who received >1 mA current and those who received <1 mA current for individualized dose stimulation. This categorization is identical to that used by Caulfield et al. (2022) on the basis of a median of E-field intensity calculated from the dose-controlled method. There was a nearly equal distribution of participants; the cohort with stimulation intensity >1 mA had 10 participants, and the other cohort had 11 participants.

10.1523/ENEURO.0374-22.2023.f8-1 Figure 8-1 The plot shows the distribution of RT for every block across active tDCS conditions—Sham, Fixed and Individualized dose. R, Random block; L, learning block. The variability in performance is similar across both conditions. For the individualized dose condition, the learning blocks 2, 3, and 4 showed a bimodal distribution, indicating a differential effect of stimulation on performance. Download Figure 8-1, TIF file.

**Figure 9.**
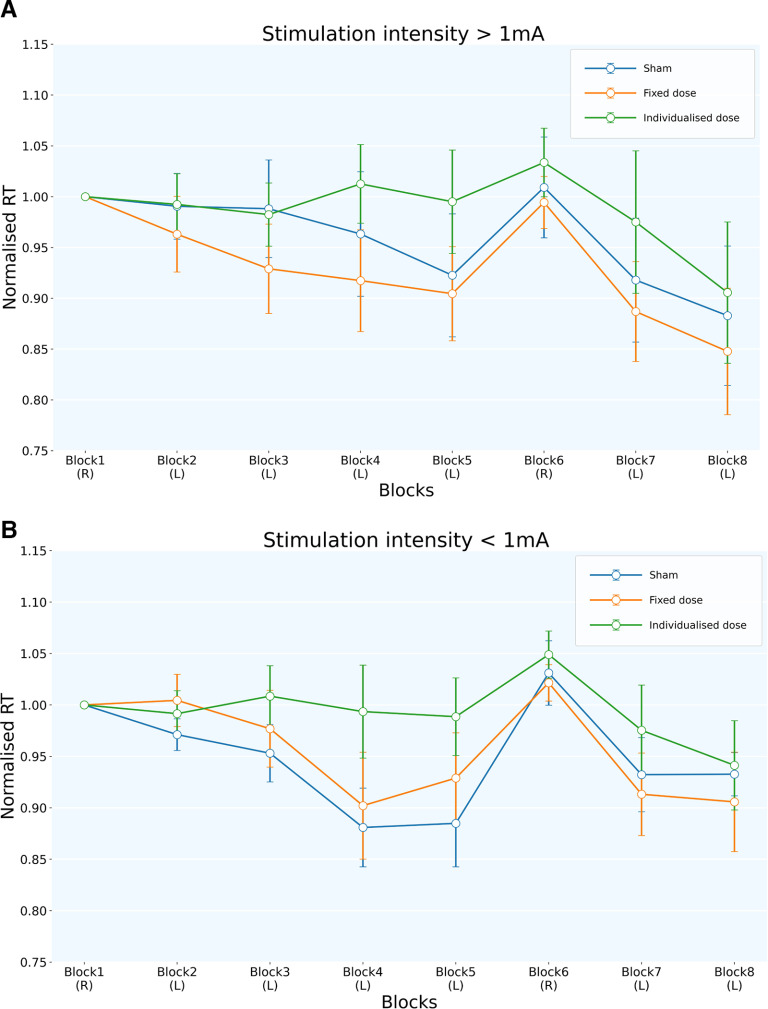
***A***, ***B***, The plots show mean RT (normalized) across blocks for different tDCS conditions for the >1 mA (***A***) and <1 mA (***B***) subject groups. R, Random block; L, learning block. The RTs across blocks are normalized to block 1. The main effect of blocks is reflected by the implicit learning trend for the sham and fixed dose conditions in the <1 mA group. This indicates that the implicit learning of the <1 mA group is better than the other group. Extended Data Figure 9-1 displays mean block-wise performance [absolute RT (ms)] across tDCS conditions. Extended Data Figure 9-2 shows the baseline performance across >1 and <1 mA groups. For block-wise correlation between the stimulation intensity and performance for the >1 and <1 mA groups, refer to Extended Data Figure 9-3.

The two-way repeated-measures ANOVA (independent variables: block and tDCS condition) revealed significant main effects of block and tDCS condition for the <1 mA group. However, the >1 mA cohort showed a significant main effect only of tDCS condition (*F*_(2,207)_ = 5.151; *p* < 0.007; η^2^ = 0.047; ANOVA by ART procedure) but not of block or a two-way interaction (Table 3). Interestingly, only the <1 mA group showed the reduced RT trend across learning blocks (main effect of block: *F*_(7,230)_ = 4.690; *p* < 0.001; η^2^ = 0.125; ANOVA by ART procedure; Table 4), reflecting implicit learning. Here again, the reducing RT (ms) trend was observed only for the sham and fixed dose conditions (main effect of condition: *F*_(2,230)_ = 4.768; *p* = 0.009; η^2^ = 0.03; ANOVA by ART procedure; Table 4). In contrast, the >1 mA group did not show the learning trend even for the sham and fixed dose conditions (Fig. 9*A*,*B*). Extended Data Figure 9-1*A*,*B* shows performance across blocks across tDCS conditions for the >1 and <1 mA groups with absolute reaction time). *Post hoc* comparisons for the main effect of conditions showed a significant difference between the sham and individualized dose condition for the <1 mA group [Individualized::Sham: *t*_(240)_ = −2.194; *p* = 0.021; Cohen’s *d* = 0.406; Tukey’s HSD; Extended Data Table 4-1]. The main effect of blocks in the <1 mA group reflects better implicit learning and performance than in the >1 mA group.

**Table 3:** Results of 2-factorial repeated-measures ANOVA for the differential effects of blocks and tDCS conditions for the >1 mA group

Term	df	df.res	Mean squared	*F* value	*p* (>*F*)	η^2^ part	Significance
Block	7	207	5561.8095	1.6144	0.1328	0.0518	
Condition	2	207	16,536.2625	5.1512	0.0066	0.0474	**
Block:condition	14	207	1236.9411	0.3783	0.9798	0.0249	

**p < 0.01.edom; df, degree of freedom, degree of freedom residuals; η^2^, eta squared (partial).

10.1523/ENEURO.0374-22.2023.tab3-1 Table 3-1. (a) *Post hoc* analysis for the main effect of blocks. The Tukey’s HSD was used for comparisons, and the resulting *p*-values were corrected for multiple comparisons using Bonferroni correction. (b) *Post hoc* analysis for the main effect of conditions. The Tukey’s HSD was used for comparisons, and the resulting *p*-values were corrected for multiple comparisons using Bonferroni correction. Download Table 3-1, DOCX file.

**Table 4:** Results of 2-factorial repeated-measures ANOVA for the differential effects of blocks and tDCS conditions for the <1mA group

Term	df	df.res	Mean squared	*F* value	*p* (>*F*)	η^2^ part	Significance
Block	7	230	20,573.6364	4.6902	0.0001	0.1249	***
Condition	2	230	22,614.2841	4.7677	0.0094	0.0398	**
Block/condition	14	230	3847.0319	0.8159	0.6515	0.0473	

**p < 0.01; ***p < 0.001. df, degree of freedom; df.res, degree of freedom residuals; η^2^, eta squared (partial).

10.1523/ENEURO.0374-22.2023.tab4-1 Table 4-1. (a) *Post hoc* analysis for the main effect of blocks. The Tukey’s HSD was used for comparisons, and the resulting *p*-values were corrected for multiple comparisons using Bonferroni correction. (b) *Post hoc* analysis for the main effect of conditions. The Tukey’s HSD was used for comparisons, and the resulting *p*-values were corrected for multiple comparisons using Bonferroni correction. Download Table 4-1, DOCX file.

**Table 5:** Results of 2-factorial repeated-measures ANOVA for the differential effects of blocks and experiment sessions

Term	df	df.res	Mean squared	*F* value	*p* (>*F*)	η^2^ part	Significance
block	7	460	106,117.7052	7.2049	3.38E-08	0.0988	***
session	2	460	361,317.1488	26.2753	1.57E-11	0.1025	***
block:session	14	460	25,725.9974	1.7607	0.0419	0.0509	*

*p < 0.05; ***p < 0.001. df, degree of freedom; df.res, degree of freedom residuals; η^2^, eta squared (partial).

10.1523/ENEURO.0374-22.2023.tab5-1 Table 5-1. (a) *Post hoc* analysis for the main effect of blocks. The Tukey’s HSD was used for comparisons, and the resulting *p*-values were corrected for multiple comparisons using Bonferroni correction. (b) *Post hoc* analysis for the main effect of sessions. The Tukey’s HSD was used for comparisons, and the resulting *p*-values were corrected for multiple comparisons using Bonferroni correction. Download Table 5-1, DOCX file.

10.1523/ENEURO.0374-22.2023.f9-1 Figure 9-1 ***A***, ***B***, The plots show the mean RT (ms) across blocks for each condition for the two cohorts. ***A*** and ***B*** represent the participants with stimulation >1 and <1 mA, respectively. Blocks 1 and 6 (marked “R”) represent the random blocks, whereas the remainder represents learning blocks. The baseline RT values for each condition for the >1 mA cohort are as follows: Sham, mean = 467.012 ± 82.93 ms; Fixed, mean = 443.712 ± 55.39 ms; Individualized, mean = 428.002 ± 89.78 ms. The baseline RT values for each condition for the <1 mA cohort are as follows: Sham, mean = 374.684 ± 65.54 ms; Fixed, mean = 380.877 ± 67.08 ms; Individualized, mean = 362.452 ± 43.03 ms. The individualized dose condition had the least RT at baseline. The error bars represent the SEM. Download Figure 9-1, TIF file.

10.1523/ENEURO.0374-22.2023.f9-2 Figure 9-2 Baseline performance. Block 1 of the sham condition is used to evaluate the baseline performance. The <1 mA group showed significantly better performance at baseline than the other group. Download Figure 9-2, TIF file.

10.1523/ENEURO.0374-22.2023.f9-3 Figure 9-3 ***A***, ***B***, Block-wise correlation between stimulation dose (mA) and the RT (ms) for the cohort with >1 mA (***A***) and <1 mA (***B***) stimulation groups. R, Random block; L, learning block. The correlation was nonsignificant for all the blocks. Download Figure 9-3, TIF file.

Further investigating these results, we compared the baseline performance of the two groups for sham condition block 1 (no active stimulation). We found a significant difference between the two groups; that is, the subjects in the <1 mA group subjects had shorter RTs than those in the >1 mA subjects (>1mA::<1mA: *t*_(17.148)_ = 2.812; *p* = 0.012; Cohen’s *d* = 1.235; Welch’s test; Extended Data Fig. 9-2). Nevertheless, both groups showed similar outcomes in the individualized dose condition (i.e., without a learning trend). Extended Data Fig. 9-3, *A* and *B*, shows block-wise Pearson’s correlation between the stimulation intensity (mA) and reaction time (ms) for the two cohorts.

#### tACS

**Figure 10.**
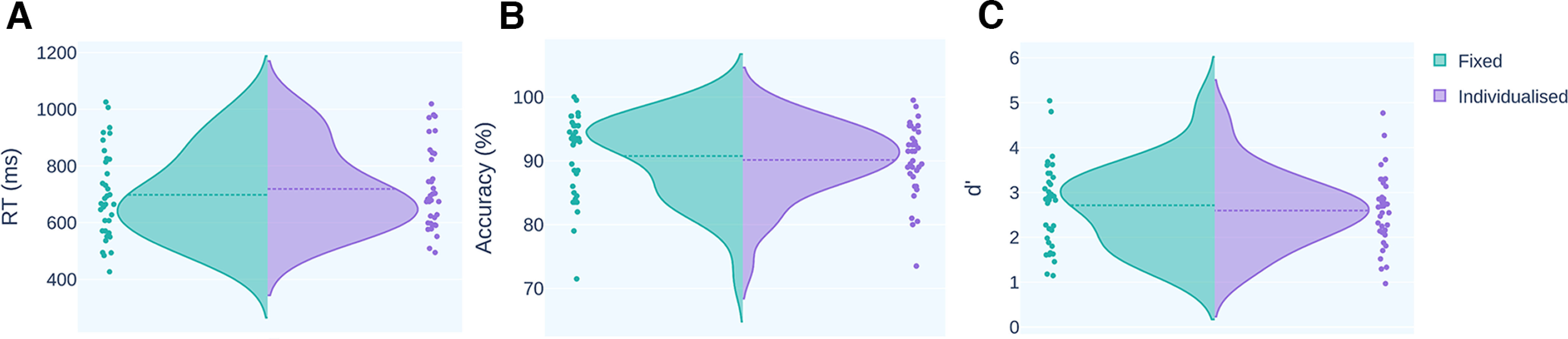
Behavioral performance across fixed and individualized dose conditions for the tACS experiment. ***A***, RT. ***B***, Accuracy. ***C***, *d*′. To review the distribution of RT (ms) across the sham condition, refer to Extended Data Figure 10-1.

We used RT, accuracy, and *d'* as the primary outcome measure of analysis (Fig. 10, Extended Data Fig. 10-1). We did not find any significant difference in performance across conditions for any of the behavioral outcomes. Also, the variability in performance showed no significant difference across the tACS conditions.

10.1523/ENEURO.0374-22.2023.f10-1 Figure 10-1 Behavioral performance across sham, fixed, and individualized dose tACS conditions. ***A***, RT. ***B***, Accuracy. ***C***, $d^{\prime }.$ Download Figure 10-1, TIF file.

For the analysis of RTs, we considered only the correct responses for analyses. The fixed dose condition had the lowest RT (mean ± SD = 698.118 ± 155.58 ms), while the individualized dose and the sham conditions had almost the same RTs (mean ± SD; Sham, 716.264 ± 166.59 ms; Individualized, 718.811 ± 144.89 ms). The one-way ANOVA showed no significant difference in RT across conditions (Q(2) = 0.4; *p* = 0.819; Kendal’s W = 0.006; Friedman test; Fig. 10*A*, Extended Data Fig. 10-1*A*).

The analysis for accuracy did not result in any significant difference across the tACS conditions (Q(2) = 0.927; *p* = 0.629; Kendal’s W = 0.013; Friedman test; Fig. 10*B*, Extended Data Fig. 10-1*B*). The mean accuracy for all the conditions was approximately the same (mean ± SD; Sham, 90.24 ± 5.41%; Fixed dose, 90.76 ± 6.44%; Individualized dose, 90.13 ± 5.62%).

Last, we did not find a significant difference of means between the d′ values across tACS conditions (Q(2) = 1.2; *p* = 0.549; Kendal’s W = 0.017; Friedman test; Fig. 10*C*, Extended Data Fig. 10-1*C*). The mean d′ was similar across tACS conditions (Sham, 2.569 ± 0.73; Fixed dose, 2.716 ± 0.94; Individualized dose, 2.599 ± 0.82).

## Discussion

In the current study, our findings show that although individualized dose tES reduces the variability in the E-field intensities at the targeted cortical surfaces considerably, it does not reduce the variability in behavioral outcomes. Moreover, we did not find a significant correlation between E-field intensity and behavioral performance in either of the behavioral tasks. These results do not support the hypothesis that physiological or behavioral effects relate linearly to the E-field intensities at a targeted cortical region. Other parameters may be equally relevant, including the current flow direction, sample size, the task performed during the session, the spatial distribution of E-field intensity, the number of stimulation sessions, or the polarization gradient across the cortical surface (Saucedo-Marquez et al., 2013; Jackson et al., 2016; Rawji et al., 2018; Biel et al., 2022).

### tDCS

Unfortunately, we could not replicate the behavioral results of past studies. In the tDCS experiment, we expected to see enhanced motor learning with fixed dose tDCS compared with the sham condition, which we did not observe. This further reiterates the issue of the inconsistent effect of tES protocols across different subject groups (Stagg et al., 2011; Wiethoff et al., 2014; Ambrus et al., 2016). Wiethoff et al. (2014) showed variability in motor evoked potentials in response to tDCS, with ∼50% of the participants showing minor or no effects of stimulation and the remaining 50% showing a facilitatory effect with both anodal and cathodal stimulation. Further, implicit learning was evident in our subjects with both fixed dose and sham stimulation conditions, although this trend was not seen with individualized dose stimulation. One potential explanation could be that an individualized dose reduces the variance in the spatial distribution of the E-fields at the cortical site as well as across the brain (Evans et al., 2020), which might, in turn, affect the excitability of the other brain areas (small-scale and large-scale networks) that could have indirectly affected the task performance (Nitsche, 2011; Yavari et al., 2017).

Probing into individual subject performances, 9 of 23 participants performed best in the sham stimulation and poorest in the individualized condition. These participants reported mild to moderate tES side effects such as tingling, itching, burning, and pain sensations in the feedback form, which could have affected their ability to concentrate on the task (Kessler et al., 2012). Another nine participants, however, showed improved task performance with the individualized dose, and, interestingly, they did not report any side effects from the stimulation. These findings suggest that the effectiveness of the individualized dose may be subject to chance, and that side effects need to be considered while designing tDCS protocols to enhance behavioral performances. Interestingly, we found a positive correlation between the individualized stimulation doses and RTs for different blocks, particularly in the randomized blocks, implying poor performance with increased stimulation dose. The gradual decrease in the correlation through the learning blocks could be because of the following two reasons: first, the participants may have learned the sequence implicitly and improved their performance; and second, they gradually became habituated to the stimulation sensation and performed better in the later blocks. Therefore, increasing stimulation intensity and E-field intensity does not necessarily correlate with improved behavioral performance. This has also been reported by others in the past (Esmaeilpour et al., 2018), implying that several factors other than current intensity influence behavioral outcomes.

Furthermore, analyzing the performance across tDCS sessions showed that participants performed significantly better in their first session than in subsequent sessions. Also, session 1 showed a clear learning trend indicating implicit motor learning across the blocks. However, since the order of the sham, fixed, and individualized tDCS conditions were randomized across participants, we think that the stronger learning effect seen in session 1 is unlikely to have influenced our results. Although studies claim that learning effects are minimal in these tasks (Willingham et al., 2000; Nitsche et al., 2003), our results show that they cannot be fully neglected.

In the exploratory *post hoc* analysis, the categorization of subjects based on stimulation intensity revealed by the bimodal distribution in performance in the individualized dose condition shows that only the participants from the <1 mA group (*N* = 11) showed the effect of learning, while the >1 mA group (*N* = 10) did not. Additionally, individualizing the stimulation dose did not reduce the interindividual performance variability. Attributes such as anatomic characteristics, genetics, age, and architecture of local inhibitory and excitatory circuits each play a role in behavioral outcomes (Li et al., 2015). The striking disparity in the performance that we observed in our study might have resulted from a few or all of these variables or possibly because of the small sample size after classification.

### tACS

In the working memory task, tACS did not significantly affect the performance for any of the outcome measures—RT, accuracy, or d′ measure of sensitivity. The trielectrode montage used for stimulating frontoparietal cortices did not deliver a uniform E-field at the corresponding brain targets. The nonreproducibility of the tACS effect with the same electrode montage on frontoparietal stimulation has also been reported previously (Kleinert et al., 2017), and there could be multiple reasons for these results. There are mixed results regarding the kind of effects seen with the use of tACS for working memory. A study by Pahor and Jaušovec (2014) has reported differential effects of frontal and parietal stimulation for fluid intelligence, with parietal stimulation of tACS showing more pronounced effects. Additionally, the dual-site tACS causes unintended stimulation of the nontargeted brain region (here, temporal region), which might have a differential role in the behavioral task (Saturnino et al., 2017; Alekseichuk et al., 2019). Another reason could be that the task was too easy for the participants, as the same results were obtained for each stimulation condition; perhaps a more difficult task like the 3-back task would have been more suitable (Biel et al., 2022). It has previously been reported in several studies that the difference in performance is found only for subjects who are poor baseline performers (Tseng et al., 2012; Li et al., 2015). This illustration also corroborates previous fMRI literature on frontoparietal activation, where the frontal and parietal cortices showed robust BOLD activity only for hard::easy contrast across tasks (Fedorenko et al., 2013).

Another reason could be that sham stimulation had a biologically meaningful effect (Fonteneau et al., 2019). Alternatively, participants could differentiate between active and sham stimulation (Greinacher et al., 2019). However, this is less likely to be the case as the stimulation intensity was 0.5 mA for each anode, and most participants did not report any side effects in the tES feedback form (e.g., itching, tingling, burning over the skin) from the tACS stimulation. Or simply, the anodal stimulation produced relatively weak E-fields in the targeted cortical region that could not produce a physiological effect (Datta et al., 2009). Nonetheless, individualizing the dose did not reduce the variability in behavioral performance. Perhaps, individualizing the dose parameter does not exclusively control the behavioral outcome. A recent study on animal models (Krause et al., 2022) reported that the baseline frequency preference of the targeted brain region determines the effect of tACS—the tACS frequency competes with the ongoing intrinsic neural oscillations to control spike timing. It first decreases the entrainment, and only at a higher stimulation amplitude does the possibility of the entrainment increase. Even a slight mismatch in the neural and stimulation frequency dramatically affects entrainment. Also, studies have found differential effects of slow and fast theta on working memory, with slow theta (4–4.5 Hz) but not fast theta (7 Hz) enhancing working memory (Bender et al., 2019; Jones et al., 2019). Another reason we could not find any significant difference between the tACS stimulation and the sham conditions could be the small sample size; the power analysis was computed assuming a larger expected effect size of at least 70 ms across tACS conditions. However, the mean difference between active and no stimulation conditions was merely 20 ms.

Thus, our findings in this study do not support a direct relationship between the E-field intensity and the behavioral output. tES affects both local and global brain networks causing neurochemical and neuroelectrical modulations in those regions; thus, contributing synergistically to the overall performance (Yavari et al., 2017). The targeted E-field (0.114 V/m) used for the tDCS experiment is relatively low compared with the E-fields (∼0.185 V/m) in studies by Evans et al. (2020) and Caulfield et al. (2022), which might have resulted because of low sample size (*n* = 23) or because of the electrode montage used in the experiment. Typically, the brain regions between the target and the reference electrodes are affected by tES (Nasseri et al., 2015; Yavari et al., 2017). Although individualized dose stimulation reduces variability in the E-field intensity at the targeted region, it fails to limit the spatial distribution of the E-fields (Evans et al., 2020), leading to the stimulation of unintended brain regions. Moreover, the electrode montage significantly adds to the interindividual variability in the amount of radial inward current (Evans et al., 2022). Another major point of concern is that the trielectrode montage for the tACS experiment did not reduce the variability in the E-fields at the frontoparietal cortices, as expected. Therefore, this experiment may not have been literally dose controlled since the pipeline for dose individualization, proposed by Evans et al. (2022), is limited to bielectrode montages. However, we extended the same pipeline to replicate tACS effects on working memory using the trielectrode montage, as shown by Violante et al. (2017). In this attempt, although the variance of E-field intensity could not be effectively reduced over both active target regions, we were able to bring the E-fields above the threshold value of 0.1 V/m, thereupon ensuring that the absence of behavioral effects was not because of low E-field intensity (Caulfield et al., 2022). This clearly proved our assumption wrong by revealing that reducing variability in E-fields over one region may not necessarily impact E-fields over other brain regions similarly. Although we used the higher of the two doses required for stimulation, in 28 of 36 subjects, the dose for the frontal electrode was always higher and hence the dose was individualized based on the frontal electrode. This could be a reason for higher variance of E-field over the parietal electrode. An ideal way to have reduced variability would have been to use different stimulation intensities over the two sites, which was technically not possible with our system. Overall, this reiterates that optimizing the electrode montage for current spread and direction for each stimulation site becomes just as crucial.

In summary, individualizing the stimulation intensity did not reduce interindividual variability in behavioral performance. In this study, we investigated whether the pipeline proposed by Evans et al. (2020) for dose individualization reduces variance in participants’ behavioral performance; the results do not support a linear relationship between the E-field and the behavioral performance. More robust analysis techniques like the Bayesian and machine learning models may provide more concrete evidence about the relationship between E-fields and tES efficacy. In accordance with recent studies, we suggest functional targeting using fMRI and tES to target the functionally relevant areas in a specific task (Jackson et al., 2016; Yavari et al., 2017; Esmaeilpour et al., 2020). In addition to the individualization of stimulation intensity (Esmaeilpour et al., 2018), the tES intervention must precede the optimization of other parameters like the number of stimulation sessions (Reis et al., 2009), the direction of current flow (Rawji et al., 2018; Evans et al., 2022), focality of the stimulation (Mikkonen et al., 2020), and baseline physiological and cognitive functions like attention (Antal et al., 2007; Biel et al., 2022) and performance ability (Tseng et al., 2012). Although dose individualization delivers a uniform E-field intensity accounting for the anatomic variability, its influence on the functional outcome in the brain is nonlinear and complex (Yavari et al., 2017). Moreover, the optimization of tES protocols to improve the effect size and clinical reproducibility necessitates a deeper understanding of the functional and physiological changes in the brain caused by tES.

## Synthesis

Reviewing Editor: Alexander Soutschek, Ludwig-Maximilians-Universitat Munchen Decisions are customarily a result of the Reviewing Editor and the peer reviewers coming together and discussing their recommendations until a consensus is reached. When revisions are invited, a fact-based synthesis statement explaining their decision and outlining what is needed to prepare a revision will be listed below. The following reviewer(s) agreed to reveal their identity: Carys Evans.

Reviewer 1

The authors set out to investigate whether delivering an individualized dose of tDCS or tACS current is superior to a standard dose in obtaining behavioral improvements. To investigate this, they designed a study to replicate and extend a study by Nitsche et al (2003) with a motor task, targeting motor cortex using anodal tDCS, as well as replicating and extending a study by Violante et al (2017) with a working memory task, targeting fronto-parietal cortices using theta tACS. The additional condition included here was a simulation condition where an individualized dose of tDCS or tACS, respectively was delivered. This was compared to a stimulation with a standard 1 mA dose and a sham protocol, as in the previous studies. The current data do not replicate findings from the two original studies:

For motor tDCS during sequence tapping, contrary to the hypothesis, RTs in the real stimulation condition did not differ statistically from the sham stimulation. And although RTs in the individual stimulation condition did significantly differ from the sham condition, this was not according to the hypothesized pattern, but because surprisingly, participants showed no behavioral effect of sequence learning in the individualized sessions (in descriptive statistics). A positive correlation between the stimulation dose and RT was found.

An exploratory analysis comparing those participants who received >1mA current and those who received <1mA current found that descriptively, this pattern of results was the same in the <1mA group, whereas the >1mA group showed no sequence learning and has slower baseline RTs to begin with.

For fronto-parietal theta tACS during working memory, as well contrary to the hypothesis, neither RTs, nor accuracy differed between real and sham stimulation conditions.

The introduction is well written and cohesively conveys the background and hypotheses that led to conducting this study. I have only one minor suggestions for the authors to consider. However, I have some concerns focusing on the presentation of methods and results that in my opinion would need to be addressed by the authors. Concerning the interpretation and discussion of results, I find that critical aspects are missing, and the discussion section may need to be rewritten before this manuscript could be accepted for publication.

Introduction:

1. The two sentences in Lines 48-54 (page 2) motivate the core argument for conducting this study, namely the poor reproducibility of tES studies and potential reasons for it. They remain a bit more vague than necessary and would benefit a lot from making it explicit that tES efficacy on what outcomes (i.e. behavior in general or specific performance in what behavioral, cognitive or motor domains?; which stimulated brain areas? short term effect or long-term effects?) were reported to show mixed results and in which specific previous studies or meta-studies these have been previously reported (i.e. giving specific references).

Methods and Results:

2. Page 4, lines 83-86: To enhance transparency, please provide a complete report of the power analyses for both experiments (if not here then please refer the interested reader to the supplemental materials), including: 1) naming the available effect sizes extracted from the Nitsche study or Violante study, respectively, 2) giving your rationale for picking the assumed effect size (which of the experiments / analyses / electrode montages from the study was taken as a basis for your power analysis and why, or was the assumed effect size averaged from several reported effect sizes from the respective study, or was the minimal effect size taken etc.); 3) naming the statistical test powered for, and 4) naming (and citing) which software package was used to estimate the minimal sample size needed.

3. Page 4. Line 9 I believe it has to be Edinburgh

4. Page 12 and Page 9: Following the current description of the data analysis, it is still unclear to me whether for the nonparametric ANOVA on normalized RT data, Block 1 was still included as a factor? Given that the data from the other blocks was normalized to it, I think one would not want to include Block 1 data after normalization anywhere, given that it would not carry any additional information anymore... but given that the description in the methods speaks of 8 factor levels for Block, and all figures show Block 1 as a factor level as well, you maybe did? Could you please clarify this in the manuscript?

5. Page 12, line 271: In what way is block 6 a random block? This is mentioned here for the first time in the main manuscript text. Could you please clarify. It might be helpful to include a minimal description of the experimental paradigm in the main manuscript text to avoid such ambiguities.

As I understood the supplemental information, then both Block 1 and Block 6 are random blocks, as in the Nitsche paper? Please include a short explanation as to why these random blocks were introduced (I expect you were most interested in the RT difference between Blocks 5 and 6 as an index for implicit sequence learning). As this is central information, I think it should be preferably included in the main manuscript text.

To make the results figures stand-alone comprehensible, it would also be good to provide this info in the figure caption or even better in the axis tick labels (e.g. instead of putting “Block 1”, Block 2”, ..., one could put “B1 random” “B2 sequence”, “B3 sequence”, ... “B6 random”, ... as axis labels), so that the reader understands the qualitative difference between blocks at first sight.

6. For the analysis of RTs in the tDCS study, you excluded incorrect trials and ultra-slow and ultra-fast responses. How many trials pe condition remained on average and as minimum and maximum?

7. In the results section, you report only p-values for the significant results, sometimes even these are missing. Please report all inference statistical results with a complete set of relevant test-scores, degrees of freedom and p-values (for example, for effects from the ANOVA: F-score, degrees of freedom, p-value). Please report standardized effect size measures if possible.

8. When reworking the results section and including the abovementioned test-scores, please also make sure to make it transparent which conclusions are based on inferential statistics, and which are based on descriptive statistics as depicted in the figures.

9. For the tACS study, the d-prime results seem to be missing.

10. As a control analysis: Are there any learning or sequence effects across sessions, i.e. when splitting the data by session instead of stimulation condition?

11. Overall, I think the visual depiction of results could be much improved by pursuing a more consistent data visualization approach:

a. Figure legends currently read different labels across figures (see for example “Individualized” vs. “ssrt_ind” vs. “ind” vs. “Ind”) but should ideally be named the same in all figures.

b. The x axis sometimes contains an overall label (e.g. “Blocks”), sometimes not, here consistency would also be preferred

c. Some plots have a white, some a colored background, here consistency would also be preferred

d. It would be very beneficial to use a consistent color scheme for the three conditions and only use these 3 colors across all figures.

e. For all figures reading “Normalized RT” on the y-axis are lacking the unit, i.e. Normalized RT (ms)

f. Using the same scale limits for normalized RT across figures and especially also for RT in the within-figure panels of figure 7 would improve readability immensely

g. Figures 8 and 10 follow a similar purpose as figures 4 and 5, namely showing the data distribution. So presenting them in the same format (e.g. all of them as the kind of distribution plots plus points like in figures 4 and 5) would be much preferred and give a more consistent picture for the reader.

h. Specific figures:

Figure 1: I believe, it has to read “session”;

Figure 8 would benefit from a better resolution, it is quite blurry

12. Formating: I find it confusing that specifically only subchapters are numbered continuously, but chapters (e.g. Intro, Methods, ...) as well as sub-subchapters (e.g. under Results ->; behavioral data analysis -> tCDS) are not. Especially the results section would, in my opinion benefit greatly from subdividing is into labeled and numbered subsections, which would also help to differentiate between planned and exploratory analyses

Interpretation of Results and discussion:

13. Overall, I’m afraid that results have been slightly overinterpreted. For example in Line 331: “ These results clearly contradict the hypothesis that physiological or behavioural effects relate linearly with the electrical field intensities at a targeted cortical region.” This is not necessarily the case. The kind of analyses presented do not really give evidence of absence, from the nonsignificant results, we can only conclude absence of evidence. This (as well as e.g. in Line 400 and many similar sentences and interpretations in the discussion) is therefore overinterpreted. I suggest to rework the discussion with this in mind.

14. For being able to draw such conclusions, additional analyses (e.g. Bayesian approaches) that can also provide evidence for the null hypothesis when they demonstrate real evidence for the null would be required. (As is, with the nonsignificant effects stemming from null-hypothesis significance testing, we might see null results, but we might as well see inconclusive results here.)

15. In the discussion, I am missing a thorough comparison of the obtained data and results with these of previous similar studies. Instead, it focuses heavily on limitations, which are quite lengthy and not always to the point (and some of them generic limitations that apply to all tES studies out there), so I would like to suggest to rework the discussion with this in mind: connecting this work with the relevant literature. Surrounding the paper by Nitsche and colleagues (2003), as well as the paper by Violante and colleagues (2017), there are a number of interesting publications which either preceded or followed their respective publications and either showed successful or failed replications of the obtained effects (so the non-significant findings from this study are in good company). Mentioning and discussing them along with the obtained results would be highly beneficial. To name some:

• Polanía, R., Nitsche, M. A., Korman, C., Batsikadze, G., & Paulus, W. (2012). The importance of timing in segregated theta phase-coupling for cognitive performance. Current Biology, 22(14), 1314-1318.

• Kleinert, M. L., Szymanski, C., & Müller, V. (2017). Frequency-unspecific effects of θ-tACS related to a visuospatial working memory task. Frontiers in human neuroscience, 11, 367.

• Biel, A. L., Sterner, E., Röll, L., & Sauseng, P. (2022). Modulating verbal working memory with fronto‐parietal transcranial electric stimulation at theta frequency: Does it work?. European Journal of Neuroscience, 55(2), 405-425.

• Reinhart, R. M., & Nguyen, J. A. (2019). Working memory revived in older adults by synchronizing rhythmic brain circuits. Nature neuroscience, 22(5), 820-827.

• Minarik, T., Berger, B., Althaus, L., Bader, V., Biebl, B., Brotzeller, F., ... & Sauseng, P. (2016). The importance of sample size for reproducibility of tDCS effects. Frontiers in human neuroscience, 10, 453.

• Greinacher, R., Buhôt, L., Möller, L., & Learmonth, G. (2019). The time course of ineffective sham‐blinding during low‐intensity (1 mA) transcranial direct current stimulation. European Journal of Neuroscience, 5

• Stagg, C. J., Jayaram, G., Pastor, D., Kincses, Z. T., Matthews, P. M., & Johansen-Berg, H. (2011). Polarity and timing-dependent effects of transcranial direct current stimulation in explicit motor learning. Neuropsychologia, 49(5), 800-804.0(8), 3380-3388.

16. The exploratory analysis in the tDCS experiment where the whole sample was split based on the observed bimodal distribution should be interpreted with great care, given that sample size per group is considerably smaller here. This would need to be discussed explicitly, but is currently missing in the discussion of these results.

17. When bringing up previous research investigating potential ethnic differences (please avoid the word race!), you remain very vague in describing their results, merely pointing to variability and not really connecting it to your specific data. I would suggest to either discuss critically how this might really be an alternative explanation for your observed findings or to leave this out.

18. Another limiting factor that in my opinion should be mentioned when discussing the results is the criticism surrounding three electrode montages as employed previously and in this study, which inevitably leads to the strongest stimulation intensities under the return electrode and not the target brain regions, for details see e.g.

• Saturnino, G. B., Madsen, K. H., Siebner, H. R., & Thielscher, A. (2017). How to target inter-regional phase synchronization with dual-site transcranial alternating current stimulation. Neuroimage, 163, 68-80.

• Alekseichuk, I., Falchier, A. Y., Linn, G., Xu, T., Milham, M. P., Schroeder, C. E., & Opitz, A. (2019). Electric field dynamics in the brain during multi-electrode transcranial electric stimulation. Nature communications, 10(1), 1-10.

Reviewer 2

The authors explored whether individualised dose-controlled tES (tDCS/tACS) can produce more consistent modulatory effects on a motor learning (SSRT) and working memory (2-back) task. Across three sessions, task performance was compared across three conditions: sham, fixed-dose (1mA), and individualised-dose tES. Modelling results indicate that inter-individual variability in E-field intensity in the regions of interest was indeed reduced by adjusting stimulator output. However, neither individualised- nor fixed-dose tDCS/tACS significantly altered behavioural task performance. It is pleasing to see proposed dose-controlled methods be evaluated experimentally. Whilst tES did not modulate behaviour, these results critically highlight that dose-controlling tES is not straightforward. Nevertheless, the manuscript would greatly benefit from clarification in some key areas.

Major comments:

Line 143. Please can the authors state how target intensities were determined, as it is presently implied rather than explicit. Were these based on the average V/m across the sample? These intensities are quite low (∼0.1V/m) when compared to peak E-field intensities (Huang et al., 2017; Opitz et al., 2016) and intensities in a cortical target (Caulfield et al., 2020; Evans et al., 2020) observed in other studies. These relatively low E-field intensities could be explained by small sample sizes, which can exacerbate inter-individual variability (i.e., by chance, lower E-field intensities may be observed in this specific population than might be observed in a different sample). Rather than determining the target intensity within a small sample, intensities would be better defined based on a larger sample representative of your population. Whilst I do not think non-significant tES effects can be explained solely based on target intensity being “too low”, the discussion should certainly explore the potential shortfall of determining target intensities from small sample sizes highlighted here.

The authors description of the regions of interest is very confusing. Firstly, in the ROI section of the Method (from line 119), please indicate which cortical target corresponds to which behavioural task. Secondly, please refer to the anatomical target throughout the paper as opposed to electrode location. Presently, the authors describe ROIs as the right middle frontal gyrus and right inferior parietal lobule, but also “targets under F4 and P4” (e.g., Line 227).

Please elaborate on why target intensities were obtained for each anode (again presumably referring to the anatomical targets MFG and IPL here and should be referred to as such). Did the authors select multiple targets for the working memory task? It is not clear to me the reasoning behind having two target regions and only dose controlling in one. A limitation of the dose-control method by Evans et al., 2020 is that it is not equipped to fix intensity at multiple cortical locations (something that could be highlighted in the discussion).

Expanding on this, please can the authors clarify whether they either a) individualised-dose based on the intensity in one ROI (e.g. MFG) and then recorded E-field intensity in both MFG & IPL, or b) individualised-dose based on whichever ROI - MFG or IPL - had the lowest intensity with a fixed-dose. If the former, this would explain why variability increased in IPL with individualised-dose, and therefore I would not call the IFG a dose-controlled target. If the latter, this would explain why individualising dose did not substantially reduce/eliminate inter-individual variability in either ROI, and therefore I would not necessarily regard this as effective dose-control of either cortical target. Figure 5 suggests that variance has not been reduced but effectively shifted (and skewed) toward higher intensities.

What was the rationale for dividing subjects into <1mA and >1mA? It seems more intuitive to divide subjects based on intensity in the cortical target as opposed to stimulator output. That said, with such a small sample (N=10/11) there may be insufficient power for this analysis. Correlation analysis between target intensity and RT may have been better suited.

Minor comments:

- Please briefly describe the behavioural tasks in the main body of the manuscript, rather than requiring the reader to search elsewhere. This is especially problematic if the referenced papers are not open access.

- Line 121: Is the ROI a sphere? Please state explicitly.

- Line 165: Approximately 15 minutes of stimulation? Please provide more detail: mean, SD, range.

- For individualised-dose, please report in prose the stimulator output applied across the sample.

- Inconsistent terminology: EFI/electric field intensity. It is recommended that the authors avoid EFI as it is not standard nomenclature and makes the manuscript less readable.

- Why were both E-field intensity (V/m) and stimulation dose (mA) included in correlation analyses? Intensity and dose effectively capture the same variance.

- I would recommend the authors reduce the number of Figures. Some figures could be combined into one (e.g. Figure 4 & supplementary Figure 4-1).

- Lines 254 onwards: p <0.000 is not accurate statistical reporting and should instead be p < 0.001. This also applies to figures.

- The authors should define timepoints of blocks 1-8 in the Method. Presumably 1 = baseline, and 2-8 are during tDCS/tACS. Presently, the term ‘blocks’ is rather ambiguous.

- Line 265: Block 1 positive correlation: If block 1 is baseline, a positive correlation between RT and individualised-dose is meaningless.

- Line 268: Please provide statistics.

- Figure 7 & 7-1 - these do not look like robust correlations. Please apply a line of best fit.

- Line 351: please can the author elaborate on what mild-moderate side effects are.

- Line 420 - the authors refer to Indian brains but did not indicate the ethnicity of the participant group in the methods, which now appears pertinent as it is referred to in the discussion. That said, it is unclear why current flow models reliant on individual anatomical MRI scans would be less accurate at predicting E-field in different ethnic groups.

## Author Response

Reviewer 1

1. The two sentences in Lines 48-54 (page 2) motivate the core argument for conducting this study, namely the poor reproducibility of tES studies and potential reasons for it. They remain a bit more vague than necessary and would benefit a lot from making it explicit that tES efficacy on what outcomes (i.e. behavior in general or specific performance in what behavioral, cognitive or motor domains?; which stimulated brain areas? short term effect or long-term effects?) were reported to show mixed results and in which specific previous studies or meta-studies these have been previously reported (i.e. giving specific references). Specific studies related to each behaviour - motor and working memory- have been cited to show non-reproducibility of tES on motor learning (tES effect on the behaviour had already been cited previously) and working memory. In addition to these, another study on schizophrenia is cited, showing tES to be effective only in a sub-group of patients. (Section: Introduction; Paragraph 2)

2. . Page 4, lines 83-86: To enhance transparency, please provide a complete report of the power analyses for both experiments (if not here then please refer the interested reader to the supplemental materials), including: 1) naming the available effect sizes extracted from the Nitsche study or Violante study, respectively, 2) giving your rationale for picking the assumed effect size (which of the experiments / analyses / electrode montages from the study was taken as a basis for your power analysis and why, or was the assumed effect size averaged from several reported effect sizes from the respective study, or was the minimal effect size taken etc.); 3) naming the statistical test powered for, and 4) naming (and citing) which software package was used to estimate the minimal sample size needed. The details of the power analysis are provided in the supplementary material. The main text now includes a reference to the supplemental material. (Section: Supplementary Tables; Table 1-1)

3. Page 4. Line 9 I believe it has to be Edinburgh This typo has now been fixed. (Section: Material and methods; Sub-section: Study overview; Paragraph 1)

4. Page 12 and Page 9: Following the current description of the data analysis, it is still unclear to me whether for the nonparametric ANOVA on normalized RT data, Block 1 was still included as a factor? Given that the data from the other blocks was normalized to it, I think one would not want to include Block 1 data after normalization anywhere, given that it would not carry any additional information anymore... but given that the description in the methods speaks of 8 factor levels for Block, and all figures show Block 1 as a factor level as well, you maybe did? Could you please clarify this in the manuscript The reviewer has indeed raised an important point. Yes, Block-1 is included in the analysis after normalisation. We followed the same pipeline as used by Nitsche et al., 2003 for analysis to compare the two results. We analysed the data with both absolute and normalised RT, keeping the independent variables the same across both conditions. Furthermore, we wanted to look at whether there is a significant difference in performance 2 between Block-1 and Block-6 (the random blocks). Therefore, Block-1 was included in the analysis. (Section: Material and methods; Sub-section: Data analysis; Sub-subsection: Serial reaction time task: Paragraph 1)

5. Page 12, line 271: In what way is block 6 a random block? This is mentioned here for the first time in the main manuscript text. Could you please clarify. It might be helpful to include a minimal description of the experimental paradigm in the main manuscript text to avoid such ambiguities. As I understood the supplemental information, then both Block 1 and Block 6 are random blocks, as in the Nitsche paper? Please include a short explanation as to why these random blocks were introduced (I expect you were most interested in the RT difference between Blocks 5 and 6 as an index for implicit sequence learning). As this is central information, I think it should be preferably included in the main manuscript text. To make the results figures stand-alone comprehensible, it would also be good to provide this info in the figure caption or even better in the axis tick labels (e.g. instead of putting “Block 1”, Block 2”, ..., one could put “B1 random” “B2 sequence”, “B3 sequence”, ... “B6 random”, ... as axis labels), so that the reader understands the qualitative difference between blocks at first sight. We are sorry that the methodological details were not clear enough. Additional information about the behavioural tasks has now been added to the main text, including the criterion for the random blocks. The motor task is called the serial reaction time task. SRTT accesses implicit motor learning while the participant performs the task. In the SRTT, the participants perform finger movements repeatedly without being aware of a sequential order. The task consists of eight blocks; blocks 2-5 and 7-8 have the same sequence (learning blocks) to be performed repeatedly. In blocks 1 and 6 (random blocks), the sequence was random. The difference between learning Block-5 and random Block-6 gives a measure of implicit learning. Block-6 counteracts the effects of routine because it follows a random pattern, and implicit motor learning could be the only likely explanation for the difference between Blocks 5 and 6. (Section: Material and methods; Sub-section: Behavioural tasks; Sub-subsection: Serial reaction time task and 2-back task) The random blocks are now marked -R in the figures to indicate a random sequence. Additionally, we have included information in the figure legend to mention random blocks from the learned block.

6. For the analysis of RTs in the tDCS study, you excluded incorrect trials and ultra slow and ultra-fast responses. How many trials per condition remained on average and as minimum and maximum? The information has now been updated in the text. (Section: Material and methods; Sub section: Data analysis; Sub-subsection: Serial reaction time task and 2-back task)

7. In the results section, you report only p-values for the significant results, sometimes even these are missing. Please report all inference statistical results with a complete set of relevant test-scores, degrees of freedom and p-values (for example, for effects from the ANOVA: F-score, degrees of freedom, p-value). Please report standardized effect size measures if possible. 3 All the p-values mentioned in the text now contain the name of the test. Also, ANOVA results follow the F-score, degrees of freedom, p-value format. (Section: Results; Sub-section: Behavioural Data analysis)

8. When reworking the results section and including the above mentioned test-scores, please also make sure to make it transparent which conclusions are based on inferential statistics, and which are based on descriptive statistics as depicted in the figures. For clarity, the statistical result also includes the name of the test along with the test score.

9. When reworking the results section and including the above mentioned test-scores, please also make sure to make it transparent which conclusions are based on inferential statistics, and which are based on descriptive statistics as depicted in the figures. For clarity, the statistical result also includes the name of the test along with the test score.

10. As a control analysis: Are there any learning or sequence effects across sessions, i.e. when splitting the data by session instead of stimulation condition? We did not observe a significant effect of Session in our data. We have added this information in the supplementary information along with the figure. A reference to this is also included in the main text. (Supplementary material; Section: Figures; Figure 6-1)

11. Overall, I think the visual depiction of results could be much improved by pursuing a more consistent data visualization approach: a. Figure legends currently read different labels across figures (see for example “Individualized” vs. “ssrt_ind” vs. “ind” vs. “Ind”) but should ideally be named the same in all figures. b. The x axis sometimes contains an overall label (e.g. “Blocks”), sometimes not, here consistency would also be preferred c. Some plots have a white, some a colored background, here consistency would also be preferred d. It would be very beneficial to use a consistent color scheme for the three conditions and only use these 3 colors across all figures. e. For all figures reading “Normalized RT” on the y-axis are lacking the unit, i.e. Normalized RT (ms) f. Using the same scale limits for normalized RT across figures and especially also for RT in the within-figure panels of figure 7 would improve readability immensely The figures have now been revised. We were careful to keep the scale and colour characteristics constant. g. Figures 8 and 10 follow a similar purpose as figures 4 and 5, namely showing the data distribution. So presenting them in the same format (e.g. all of them as the kind of distribution plots plus points like in figures 4 and 5) would be much preferred and give a more consistent picture for the reader. Your recommendation to provide the data in this format is what we followed. The figure, however, no longer includes information for sham stimulation and only depicts active stimulation events. 4 h. Specific figures: Figure 1: I believe, it has to read “session”; The typo in the figure has now been fixed. Figure 8 would benefit from a better resolution, it is quite blurry An updated version of Figure-8 is now included.

12. Formating: I find it confusing that specifically only subchapters are numbered continuously, but chapters (e.g. Intro, Methods, ...) as well as sub-subchapters (e.g. under Results -> behavioral data analysis -> tCDS) are not. Especially the results section would, in my opinion benefit greatly from subdividing is into labeled and numbered subsections, which would also help to differentiate between planned and exploratory analyses All the sections of the text are now numbered.

Interpretation of Results and discussion:

13. Overall, I’m afraid that results have been slightly overinterpreted. For example in Line 331: “ These results clearly contradict the hypothesis that physiological or behavioural effects relate linearly with the electrical field intensities at a targeted cortical region.” This is not necessarily the case. The kind of analyses presented do not really give evidence of absence, from the nonsignificant results, we can only conclude absence of evidence. This (as well as e.g. in Line 400 and many similar sentences and interpretations in the discussion) is therefore overinterpreted. I suggest to rework the discussion with this in mind. We value the feedback and criticism you have provided. Now that the material has undergone a comprehensive second analysis, we have added and changed the sentences that state conflict with the absence of evidence.

14. For being able to draw such conclusions, additional analyses (e.g. Bayesian approaches) that can also provide evidence for the null hypothesis when they demonstrate real evidence for the null would be required. (As is, with the nonsignificant effects stemming from null-hypothesis significance testing, we might see null results, but we might as well see inconclusive results here.) This has now been added to our results as a restriction and a potential future development. (Section: Discussions; Paragraph: Last)

15. In the discussion, I am missing a thorough comparison of the obtained data and results with these of previous similar studies. Instead, it focuses heavily on limitations, which are quite lengthy and not always to the point (and some of them generic limitations that apply to all tES studies out there), so I would like to suggest to rework the discussion with this in mind: connecting this work with the relevant literature. Surrounding the paper by Nitsche and colleagues (2003), as well as the paper by Violante and colleagues (2017), there are a number of interesting publications which either preceded or followed their respective publications and either showed successful or failed replications of the obtained effects (so the non- 5 significant findings from this study are in good company). Mentioning and discussing them along with the obtained results would be highly beneficial. To name some: • Polanía, R., Nitsche, M. A., Korman, C., Batsikadze, G., & Paulus, W. (2012). The importance of timing in segregated theta phase-coupling for cognitive performance. Current Biology, 22(14), 1314-1318. • Kleinert, M. L., Szymanski, C., & Müller, V. (2017). Frequency-unspecific effects of θ tACS related to a visuospatial working memory task. Frontiers in human neuroscience, 11, 367. • Biel, A. L., Sterner, E., Röll, L., & Sauseng, P. (2022). Modulating verbal working memory with fronto‐parietal transcranial electric stimulation at theta frequency: Does it work?. European Journal of Neuroscience, 55(2), 405-425. • Reinhart, R. M., & Nguyen, J. A. (2019). Working memory revived in older adults by synchronizing rhythmic brain circuits. Nature neuroscience, 22(5), 820-827. • Minarik, T., Berger, B., Althaus, L., Bader, V., Biebl, B., Brotzeller, F., ... & Sauseng, P. (2016). The importance of sample size for reproducibility of tDCS effects. Frontiers in human neuroscience, 10, 453. • Greinacher, R., Buhôt, L., Möller, L., & Learmonth, G. (2019). The time course of ineffective sham‐blinding during low‐intensity (1 mA) transcranial direct current stimulation. European Journal of Neuroscience, 5 • Stagg, C. J., Jayaram, G., Pastor, D., Kincses, Z. T., Matthews, P. M., & Johansen Berg, H. (2011). Polarity and timing-dependent effects of transcranial direct current stimulation in explicit motor learning. Neuropsychologia, 49(5), 800-804.0(8), 3380- 3388. We appreciate your efforts in supplying the research article so that we can compare our findings with it in more detail and also support our results. We have now included a thorough comparison of our behavioural findings to the earlier findings. (Section: Discussions; Subsection: tACS; Paragraph 2,3 and 4).

16. The exploratory analysis in the tDCS experiment where the whole sample was split based on the observed bimodal distribution should be interpreted with great care, given that sample size per group is considerably smaller here. This would need to be discussed explicitly, but is currently missing in the discussion of these results. There is explicit mention of sample sizes and the explanation of why the sample was split. Furthermore, the two samples are analysed separately for the independent variables - Blocks and tDCS conditions. Details on ANOVA are added in the supplementary materials. The reader is prompted to refer to supplementary results in the main text. (Section: Results; Sub-section: tDCS; Sub-subsection: Differential effects of stimulation intensity on performance) (Supplementary tables- 4 - 9)

17. When bringing up previous research investigating potential ethnic differences (please avoid the word race!), you remain very vague in describing their results, merely pointing to variability and not really connecting it to your specific data. I would suggest to either discuss critically how this might really be an alternative explanation for your observed findings or to leave this out. We deduced from your comment and internal discussion that the current-flow modelling literature lacked conclusive and convincing information on how ethnic differences may 6 influence current-flow modelling. As a result, we have omitted this discussion in the main text.

18. Another limiting factor that in my opinion should be mentioned when discussing the results is the criticism surrounding three electrode montages as employed previously and in this study, which inevitably leads to the strongest stimulation intensities under the return electrode and not the target brain regions, for details see e.g. • Saturnino, G. B., Madsen, K. H., Siebner, H. R., & Thielscher, A. (2017). How to target inter-regional phase synchronization with dual-site transcranial alternating current stimulation. Neuroimage, 163, 68-80. • Alekseichuk, I., Falchier, A. Y., Linn, G., Xu, T., Milham, M. P., Schroeder, C. E., & Opitz, A. (2019). Electric field dynamics in the brain during multi-electrode transcranial electric stimulation. Nature communications, 10(1), 1-10. The information has now been added as the limitation to dual-site tACS protocol with appropriate citations.(Section: Discussions; Sub-section: tACS; Paragraph 2)

Reviewer 2

Line 143. Please can the authors state how target intensities were determined, as it is presently implied rather than explicit. Were these based on the average V/m across the sample? These intensities are quite low (∼0.1V/m) when compared to peak E-field intensities (Huang et al., 2017; Opitz et al., 2016) and intensities in a cortical target (Caulfield et al., 2020; Evans et al., 2020) observed in other studies. These relatively low E-field intensities could be explained by small sample sizes, which can exacerbate inter-individual variability (i.e., by chance, lower E-field intensities may be observed in this specific population than might be observed in a different sample). Rather than determining the target intensity within a small sample, intensities would be better defined based on a larger sample representative of your population. Whilst I do not think non significant tES effects can be explained solely based on target intensity being “too low”, the discussion should certainly explore the potential shortfall of determining target intensities from small sample sizes highlighted here. The Dose Individualisation subsection of the Methods section describes the process for calculating the target intensities. Because of the short sample size (Here, 23 for left-M1, 36 for frontoparietal cortices)., there may be a low electric field generation in the targeted cortices. We cannot, however, rule out the notion that the target site’s low electric field generation was a result of ethnic diversity. This hypothesis is further supported by a study that claims ethnic disparities cause variances in cortical excitability. (Section: Material and methods; Sub-section: Dose individualisation) The authors description of the regions of interest is very confusing. Firstly, in the ROI section of the Method (from line 119), please indicate which cortical target corresponds to which behavioural task. Secondly, please refer to the 7 anatomical target throughout the paper as opposed to electrode location. Presently, the authors describe ROIs as the right middle frontal gyrus and right inferior parietal lobule, but also “targets under F4 and P4” (e.g., Line 227). The ROI had a spherical shape with a 5mm radius. This fact is now explicitly mentioned in the text under the Method’s section on Regions of interest. We revised the main text to reflect your suggestion and refer to ROI as the anatomical target throughout. (Section: Material and methods; Sub-section: current flow modelling; Subsub-section: Regions of interests (ROIs)) Please elaborate on why target intensities were obtained for each anode (again presumably referring to the anatomical targets MFG and IPL here and should be referred to as such). Did the authors select multiple targets for the working memory task? It is not clear to me the reasoning behind having two target regions and only dose controlling in one. A limitation of the dose-control method by Evans et al., 2020 is that it is not equipped to fix intensity at multiple cortical locations (something that could be highlighted in the discussion). Expanding on this, please can the authors clarify whether they either a) individualised-dose based on the intensity in one ROI (e.g. MFG) and then recorded E-field intensity in both MFG & IPL, or b) individualised-dose based on whichever ROI - MFG or IPL - had the lowest intensity with a fixed-dose. If the former, this would explain why variability increased in IPL with individualised dose, and therefore I would not call the IFG a dose-controlled target. If the latter, this would explain why individualising dose did not substantially reduce/eliminate inter-individual variability in either ROI, and therefore I would not necessarily regard this as effective dose-control of either cortical target. Figure 5 suggests that variance has not been reduced but effectively shifted (and skewed) toward higher intensities. Evans’ pipeline is only relevant to bi-electrode montage, The individualised dose generated a variable electric field and thus, the procedure can’t be called dose controlled. But we still wanted to use it to explore the TACS effect on working memory performance, as demonstrated in Violante’s study. We have outlined the process and discussed the approach’s limitations. Additionally, we cited research using identical tACS electrode montage with either 1 or 2mA and contrasted our findings with those investigations. (Section: Discussion; Subsection: tACS; Paragraph 3) What was the rationale for dividing subjects into 1mA? It seems more intuitive to divide subjects based on intensity in the cortical target as opposed to stimulator output. That said, with such a small sample (N=10/11) there may be insufficient power for this analysis. Correlation analysis between target intensity and RT may have been better suited. We used a fixed dose of 1mA for the experiment. Dose-individualisation for different participants resulted in doses that were either less than or greater than the 1mA. There is no consensus on the dose intensity to be used for the tES experiment. Secondly, we could see a bimodal distribution of performance only for the individualised dose condition, indicating a dose-dependent effect. This made it clear to split the individuals into groups according to whether they received 1mA or more or less. (Section: Results; Sub-section: Behavioural data analysis; Sub-subsection: 8 tDCS; Differential effects of stimulation intensity on performance.) Correlation analysis: Supplementary Figure: 9-3(A), 9-3(B) Please briefly describe the behavioural tasks in the main body of the manuscript, rather than requiring the reader to search elsewhere. This is especially problematic if the referenced papers are not open access. Now, a brief description of the behavioural tasks is present in the main text. (Section: Material and methods; Sub-section: Behavioural tasks) - Line 121: Is the ROI a sphere? Please state explicitly. Now it is explicitly mentioned in the text that ROI is a sphere with a 5mm radius.(Section: Material and methods; Sub-section: current flow modelling; Sub subsection: Regions of interests (ROIs)) - Line 165: Approximately 15 minutes of stimulation? Please provide more detail: mean, SD, range. This information is now added and has also been revised (∼17 {plus minus} 1.95 minutes) (Section: Material and methods; Sub-section: tES; Sub-subsection: tDCS and tACS). - For individualised-dose, please report in prose the stimulator output applied across the sample. This information has now been added in the Result section. (Section: Results; Subsection: current flow modelling) - Inconsistent terminology: EFI/electric field intensity. It is recommended that the authors avoid EFI as it is not standard nomenclature and makes the manuscript less readable. Now, the term “E-field intensity” is used throughout the text. - Why were both E-field intensity (V/m) and stimulation dose (mA) included in correlation analyses? Intensity and dose effectively capture the same variance. E-field intensity and the stimulation dose had a differential effect on implicit motor learning. The correlation analysis showed significant positive correlation between the RT and Dose, whereas there was a non-significant negative correlation between E field and RT. Although the E-field and RT showed similar correlation across blocks. The Stimulation Dose vs RT showed a trend of decreasing Pearsons’ r across blocks. (Figure-7; Section: Results; Subsection: Behavioural data analysis; Sub-subsection: tDCS; Paragraph 2) - I would recommend the authors reduce the number of Figures. Some figures could be combined into one (e.g. Figure 4 & supplementary Figure 4-1). Revised figures with more consistent colour and scale characteristics have been generated and added to the text. - Lines 254 onwards: p < 0.001. This also applies to figures. All the statistical reportings now follow the recommended format. 9 - The authors should define timepoints of blocks 1-8 in the Method. Presumably 1 = baseline, and 2-8 are during tDCS/tACS. Presently, the term ‘blocks’ is rather ambiguous. We would like to clarify that stimulation was applied throughout all blocks from 1-8. Blocks 1 and 6 comprised random sequences, while the rest of the blocks had a specific learning sequence. We have now indicated this on the figures for better clarity. Block 1 is indeed the baseline which was used to normalize the performance in other blocks. - Line 265: Block 1 positive correlation: If block 1 is baseline, a positive correlation between RT and individualised-dose is meaningless. As stated above, stimulation was on for all blocks (1-8) and only the sequence was different. Hence, correlation between RT and dose would still be appropriate for Block 1. The information is explicitly mentioned in the main text @ Section: Material and methods; Subsection: Data analysis; Sub-subsection: Serial reaction time task - Line 268: Please provide statistics. The ANOVA results now follow the format [F(dof1, dof2) = ; p-value = ; name of test]. Detailed anova tables and multiple comparisons are provided in the supplementary information. - Figure 7 & 7-1 - these do not look like robust correlations. Please apply a line of best fit. A line of best-fit is now added in the correlation figures. (Figure 7, 7-1, 9-3(A), 9-3(B)) - Line 351: please can the author elaborate on what mild-moderate side effects are. The participants were asked to fill out the tES feedback form after every session. They were asked to checkmark the intensity of sensation for itching, pain, burning, warmth/heat, iron taste, and fatigue/decreased alertness felt due to tES as follows: None - I did not feel the sensation addressed Mild - I mildly felt the sensation addressed Moderate - I felt the sensation addressed Strong - I felt the sensation addressed to a considerable degree This information is now added at the end of supplementary materials. - Line 420 - the authors refer to Indian brains but did not indicate the ethnicity of the participant group in the methods, which now appears pertinent as it is referred to in the discussion. That said, it is unclear why current flow models reliant on individual anatomical MRI scans would be less accurate at predicting E-field in different ethnic groups. 10 We could not find strong and convincing evidence in the literature to show that ethnic differences may influence current-flow modelling. We have therefore omitted this discussion from the main text. This is also what another reviewer suggested.
