# Supplementary figures and images for: Behavioral Validation of Individualized Low-Intensity Transcranial Electrical Stimulation (tES) Protocols
Source: eNeuro. 2023 Dec 5;10(12):ENEURO.0374-22.2023. doi: 10.1523/ENEURO.0374-22.2023 (PMC10748339; doi:10.1523/ENEURO.0374-22.2023)

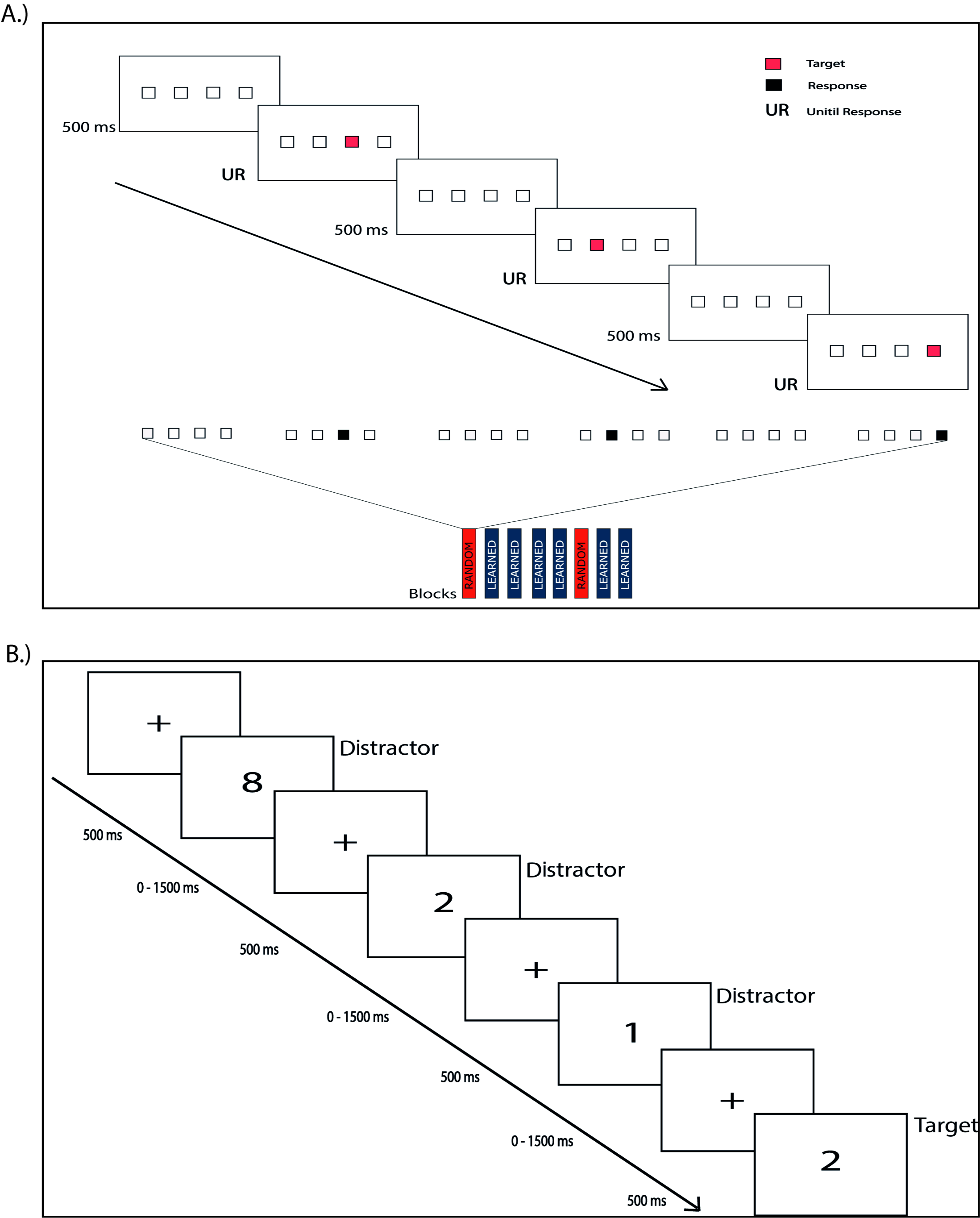

Supplement: Figure 1-1 — A, The SRTT. Each trial displayed four outlined boxes and a target, which is a solid red square. The target changed its position every trial over each box. The subjects were asked to place their right-hand index, middle, ring, and little finger over the keys labeled 1, 2, 3, and 4, respectively. They registered their response by pressing the key corresponding to the target position and the next target appeared 500 ms later. The task consisted of 8 blocks with 120 trials each. The target was presented in a pseudorandom fashion in blocks 1 and 6 in equal frequency over each position, without appearing consecutively at the same position. The rest of the blocks followed the same sequence of 12 trials (e.g., 341212342341) repeated 10 times. Participants performed 20 practice trials at the start of the first session, with visual feedback provided at the end of each trial. Subjects were unaware of the repeating sequence, and a unique sequence was used for every participant for every session. B, The 2-back task. A sequence of numbers, ranging from 0 to 9, was presented one at a time in a randomized order. The subjects were asked to register the response using their right index finger when the currently displayed number matched the one presented two trials earlier by pressing the left mouse button. The sequence consisted of 200 trials with 50 targets and 150 distractors (nontarget number). Download Figure 1-1, TIF file. [file enu-eN-NRS-0374-22-s01.tif]

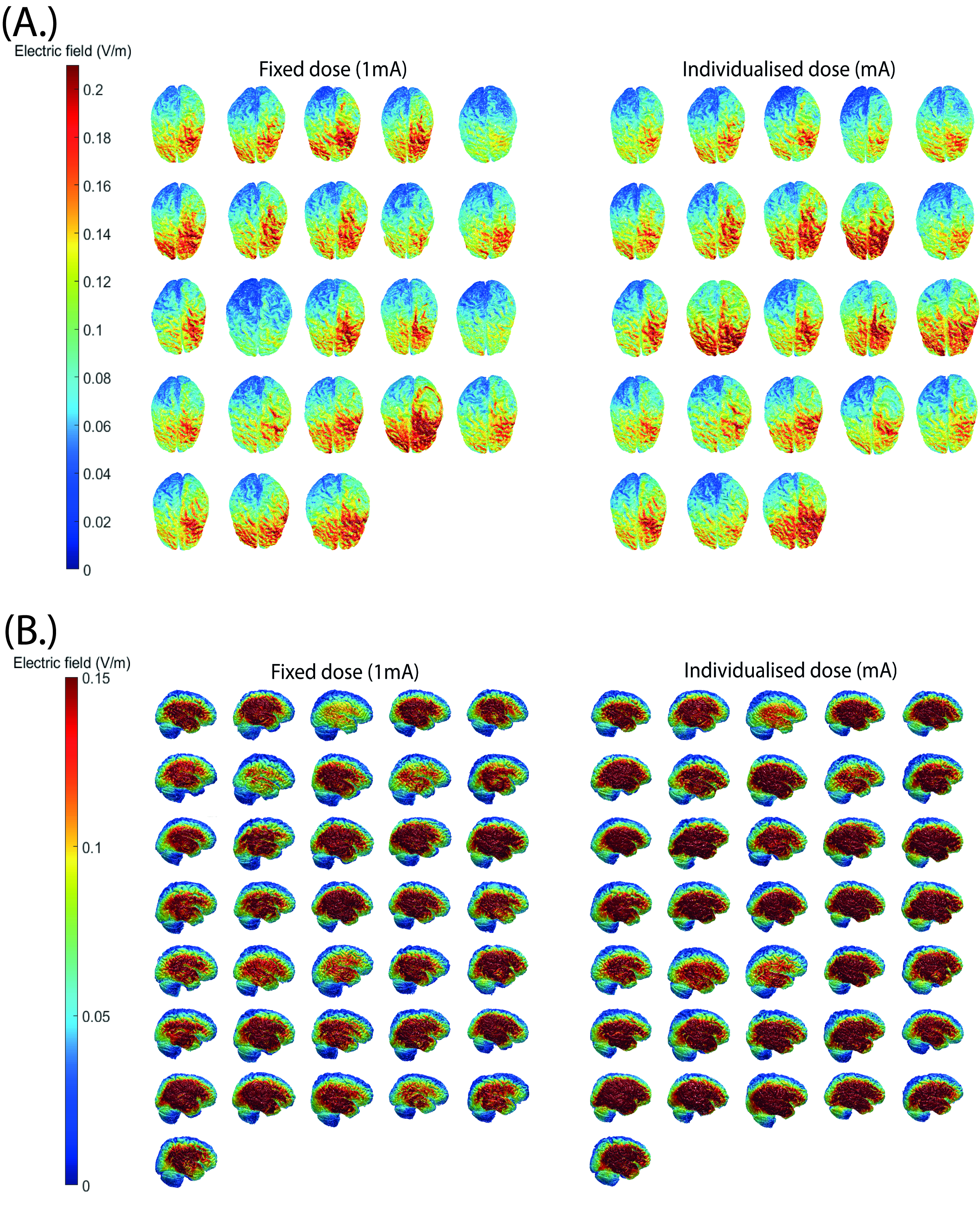

Supplement: Figure 4-1 — A, B, The figures show the qualitative comparison of the electric field generated over the left-M1 (A) and frontoparietal cortex (B). For the subjects with low electric field intensity in the fixed dose condition, the individualized dose increased the electric field to approximately the mean electric field. In contrast, the electric field was decreased for the subjects with high electric field generation in the fixed dose condition. Download Figure 4-1, TIF file. [file enu-eN-NRS-0374-22-s02.tif]

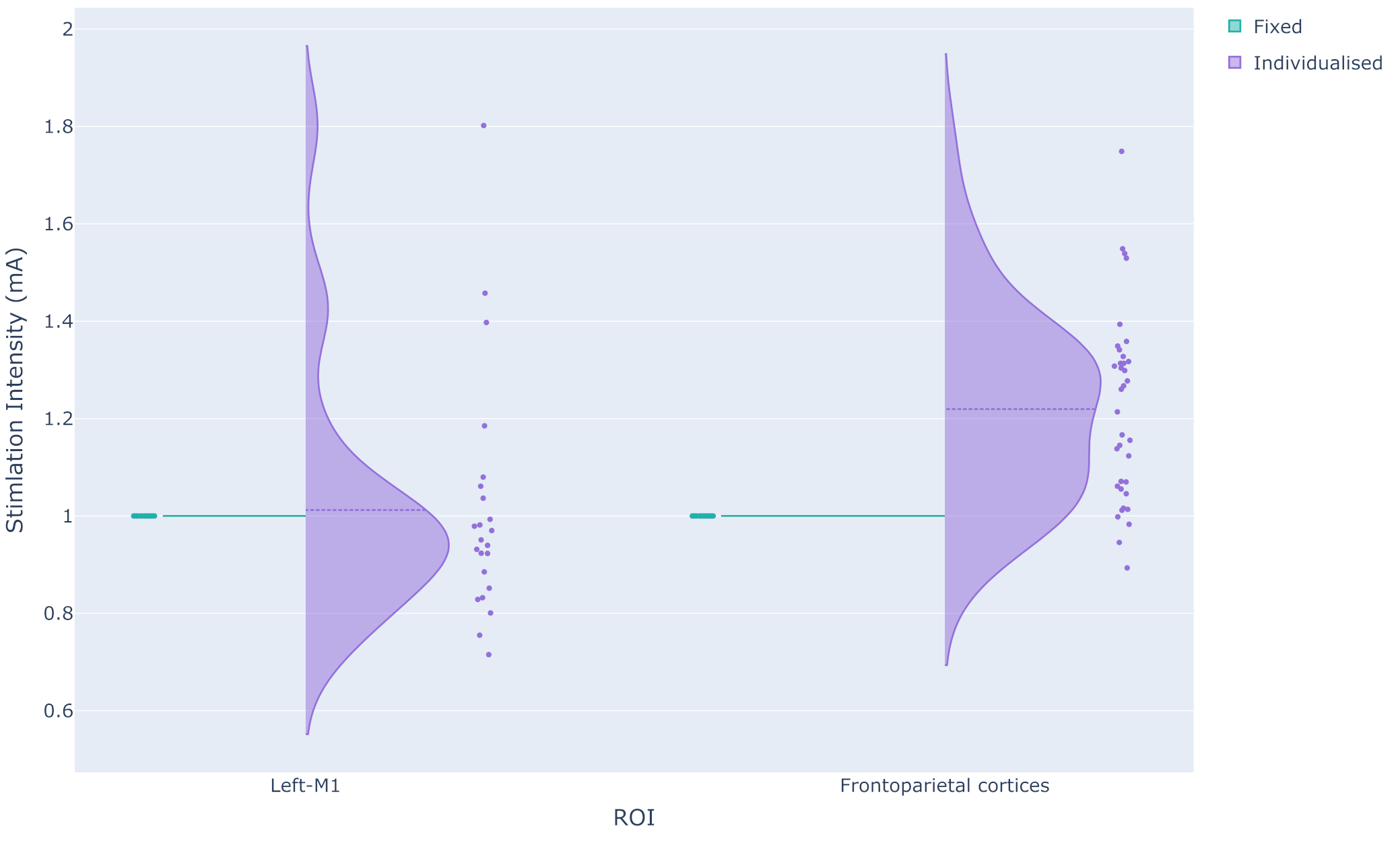

Supplement: Figure 4-2 — The stimulator output (mA) distribution for electric field intensity (V/m) in left-M1 and frontoparietal cortex. Download Figure 4-2, TIF file. [file enu-eN-NRS-0374-22-s03.tif]

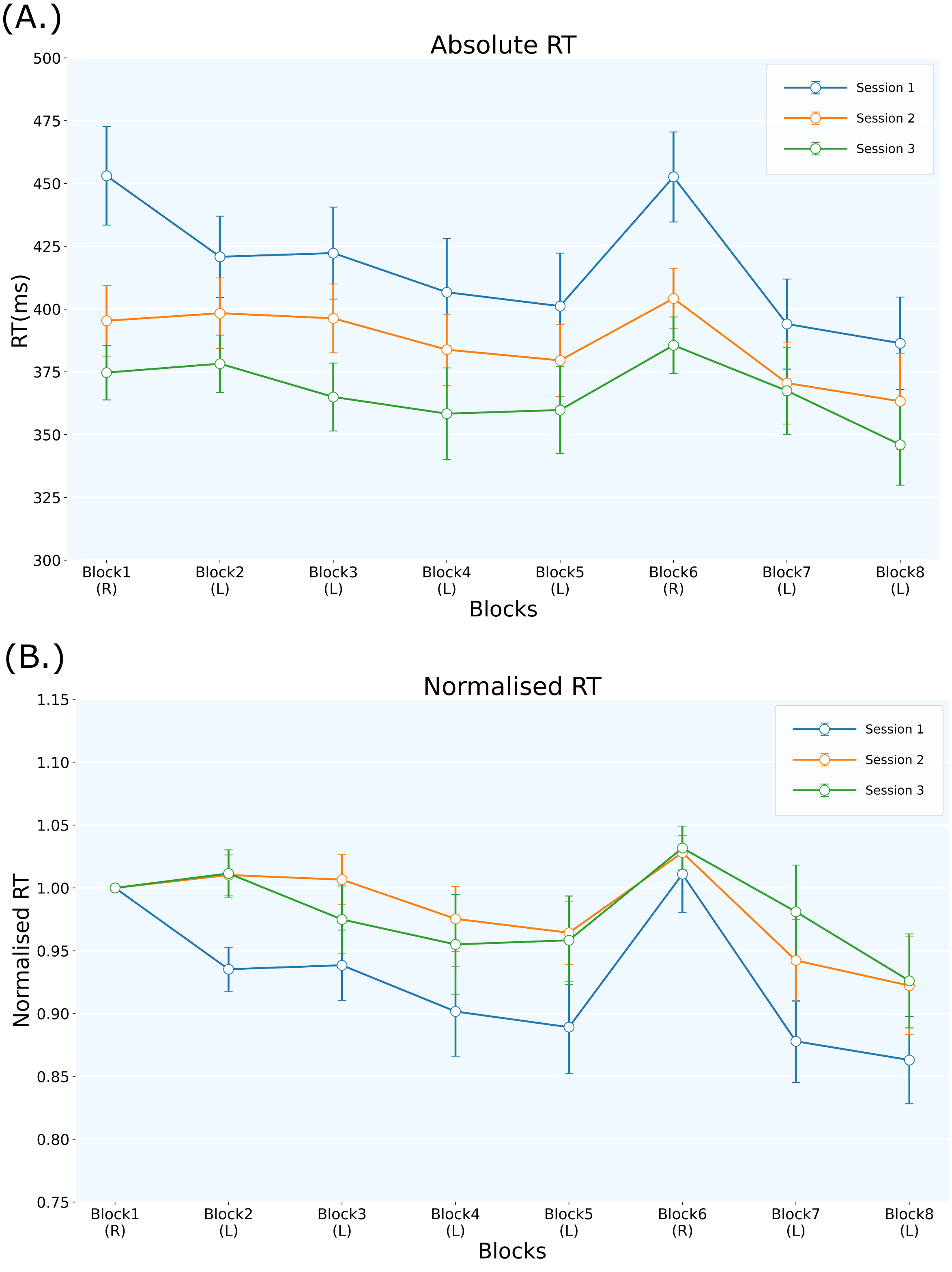

Supplement: Figure 6-1 — The figure shows participants’ performances across tDCS sessions. The y-axis represents absolute RT (ms) and normalized RT in A and B, respectively. R, Random block; L, learning block. A, The absolute RT (ms) showed a significant main effect of blocks (F(7,460) = 7.589; p < 0.001; η2 = 0.104), and sessions (F(2,460) = 64.654; p < 0.001; η2 = 0.219), but no interaction. Participants’ performances at session 1 was significantly better than at the subsequent sessions. However, the performance in sessions 2 and 3 was not significantly different. B, The normalized RTs showed a significant main effect of blocks (F(7,460) = 7.204; p < 0.001; η2 = 0.099), sessions (F(2,460) = 26.275; p < 0.001; η2 = 0.102), and also interaction (F(14,460) = 1.7607; p = 0.042; η2 = 0.051). The results indicate a significant difference in participants’ performances between the first session and the subsequent sessions. However, there was no significant difference in performances between the second and third sessions. Download Figure 6-1, TIF file. [file enu-eN-NRS-0374-22-s04.tif]

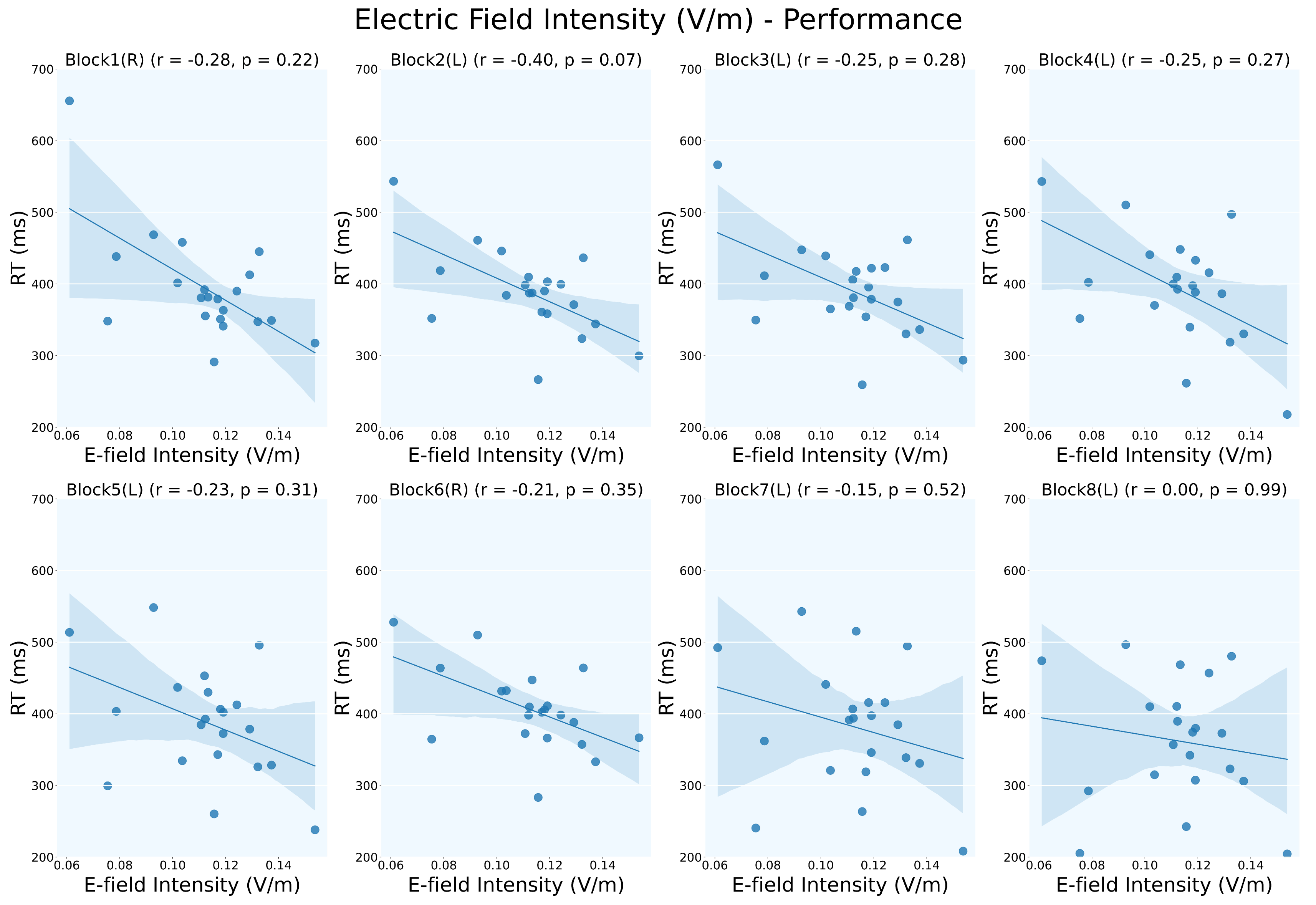

Supplement: Figure 7-1 — The graph shows the Pearson’s correlation between the electric field intensity (V/m) and the mean RTs (ms) for each block in the fixed dose condition for 21 subjects. R, Random block; L, learning block. The electric field intensity and the RT have a nonsignificant negative correlation (p > 0.05; Pearson's correlation) for all the blocks. The electric field intensity was computed from left M1 for a fixed dose of 1 mA tDCS stimulation. Download Figure 7-1, TIF file. [file enu-eN-NRS-0374-22-s05.tif]

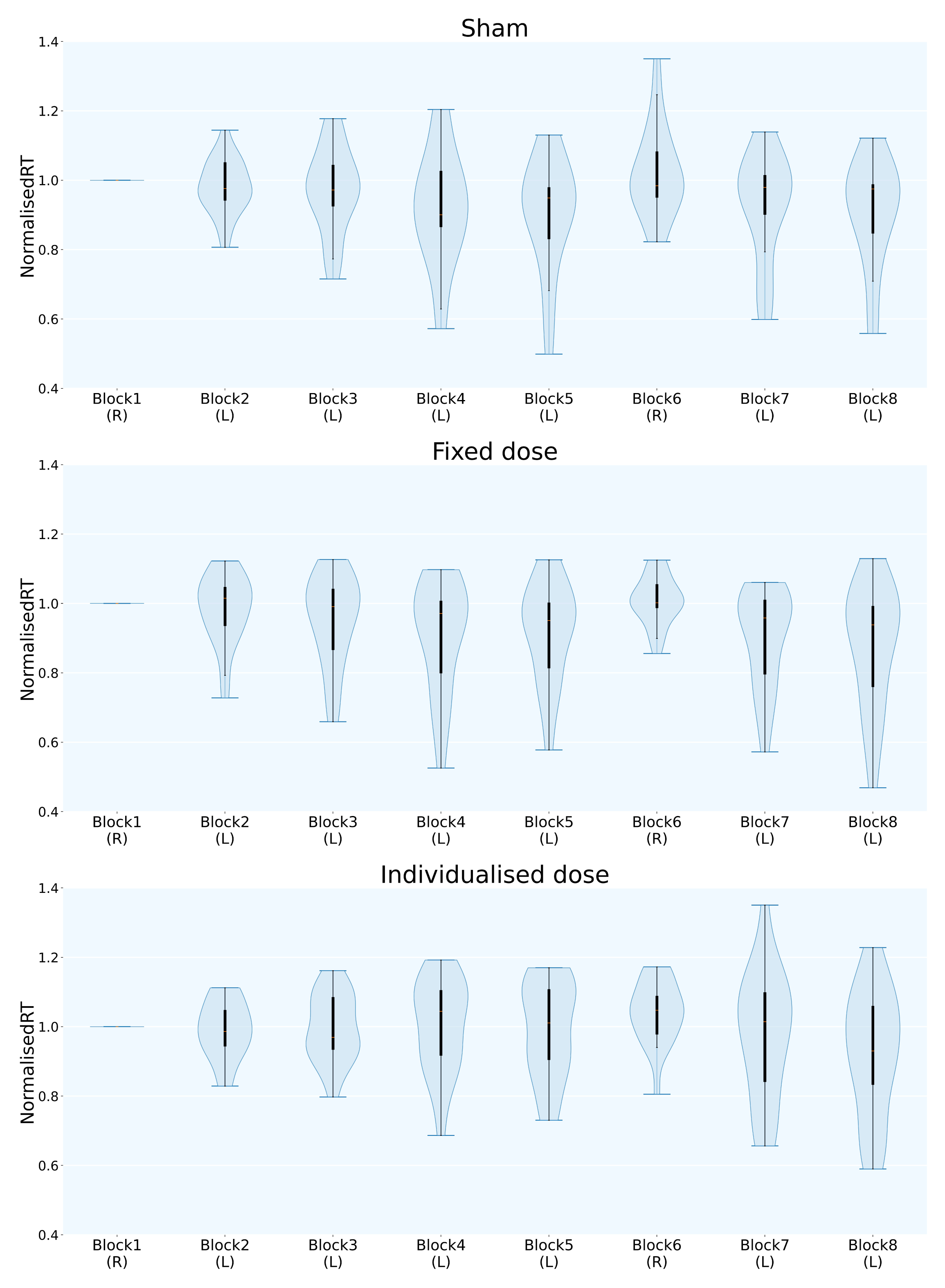

Supplement: Figure 8-1 — The plot shows the distribution of RT for every block across active tDCS conditions—Sham, Fixed and Individualized dose. R, Random block; L, learning block. The variability in performance is similar across both conditions. For the individualized dose condition, the learning blocks 2, 3, and 4 showed a bimodal distribution, indicating a differential effect of stimulation on performance. Download Figure 8-1, TIF file. [file enu-eN-NRS-0374-22-s06.tif]

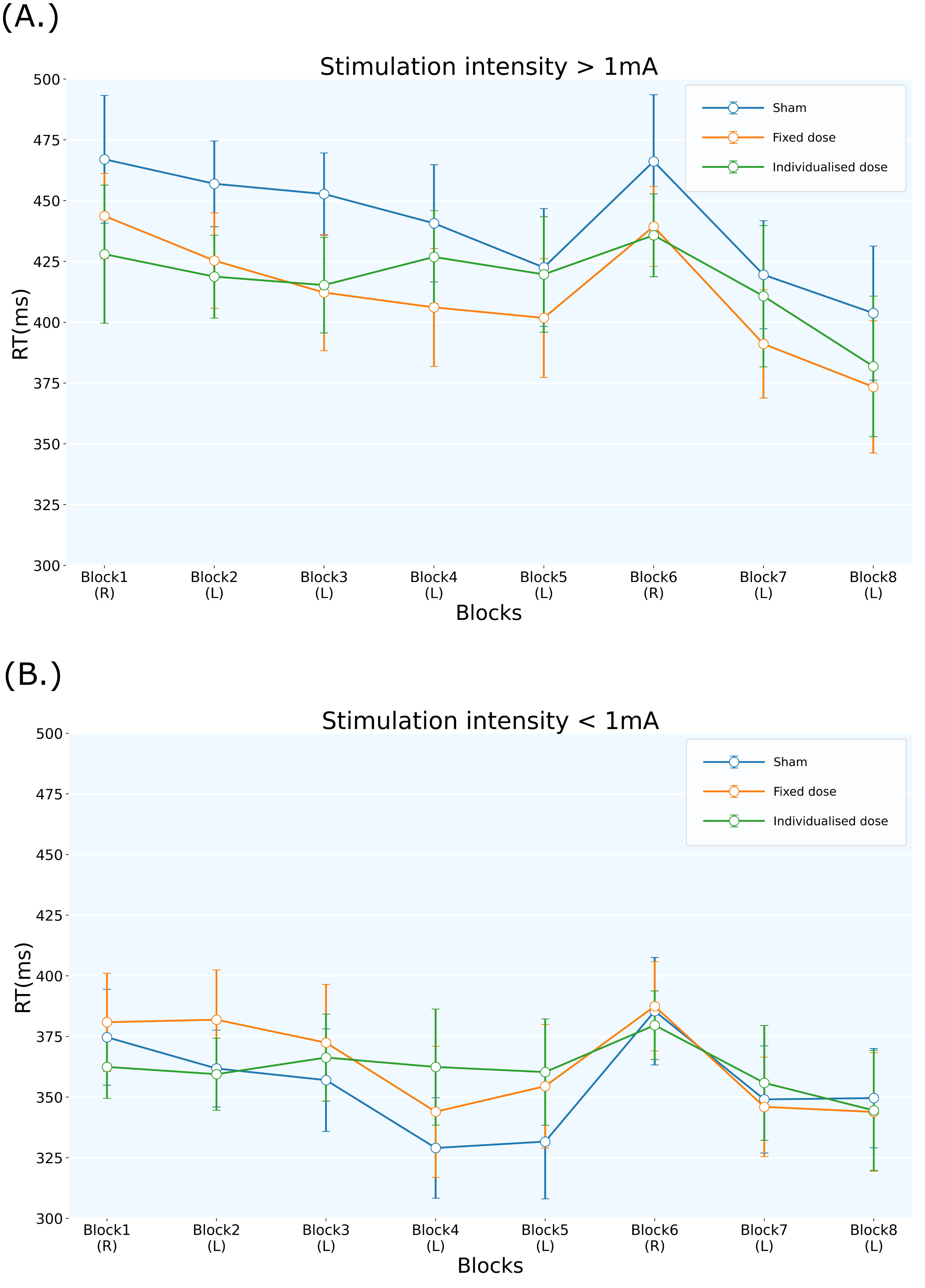

Supplement: Figure 9-1 — A, B, The plots show the mean RT (ms) across blocks for each condition for the two cohorts. A and B represent the participants with stimulation >1 and <1 mA, respectively. Blocks 1 and 6 (marked “R”) represent the random blocks, whereas the remainder represents learning blocks. The baseline RT values for each condition for the >1 mA cohort are as follows: Sham, mean = 467.012 ± 82.93 ms; Fixed, mean = 443.712 ± 55.39 ms; Individualized, mean = 428.002 ± 89.78 ms. The baseline RT values for each condition for the <1 mA cohort are as follows: Sham, mean = 374.684 ± 65.54 ms; Fixed, mean = 380.877 ± 67.08 ms; Individualized, mean = 362.452 ± 43.03 ms. The individualized dose condition had the least RT at baseline. The error bars represent the SEM. Download Figure 9-1, TIF file. [file enu-eN-NRS-0374-22-s07.tif]

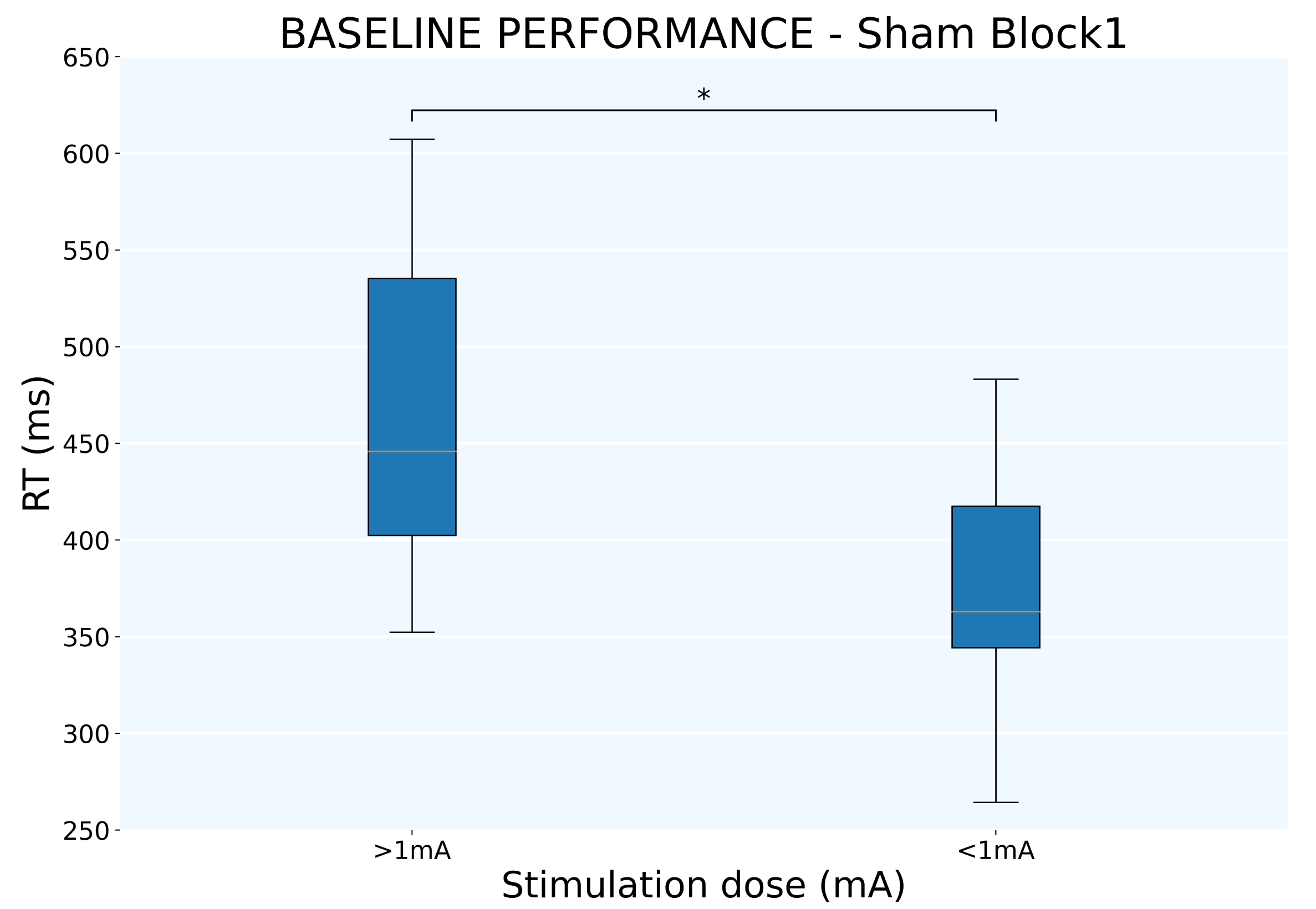

Supplement: Figure 9-2 — Baseline performance. Block 1 of the sham condition is used to evaluate the baseline performance. The <1 mA group showed significantly better performance at baseline than the other group. Download Figure 9-2, TIF file. [file enu-eN-NRS-0374-22-s08.tif]

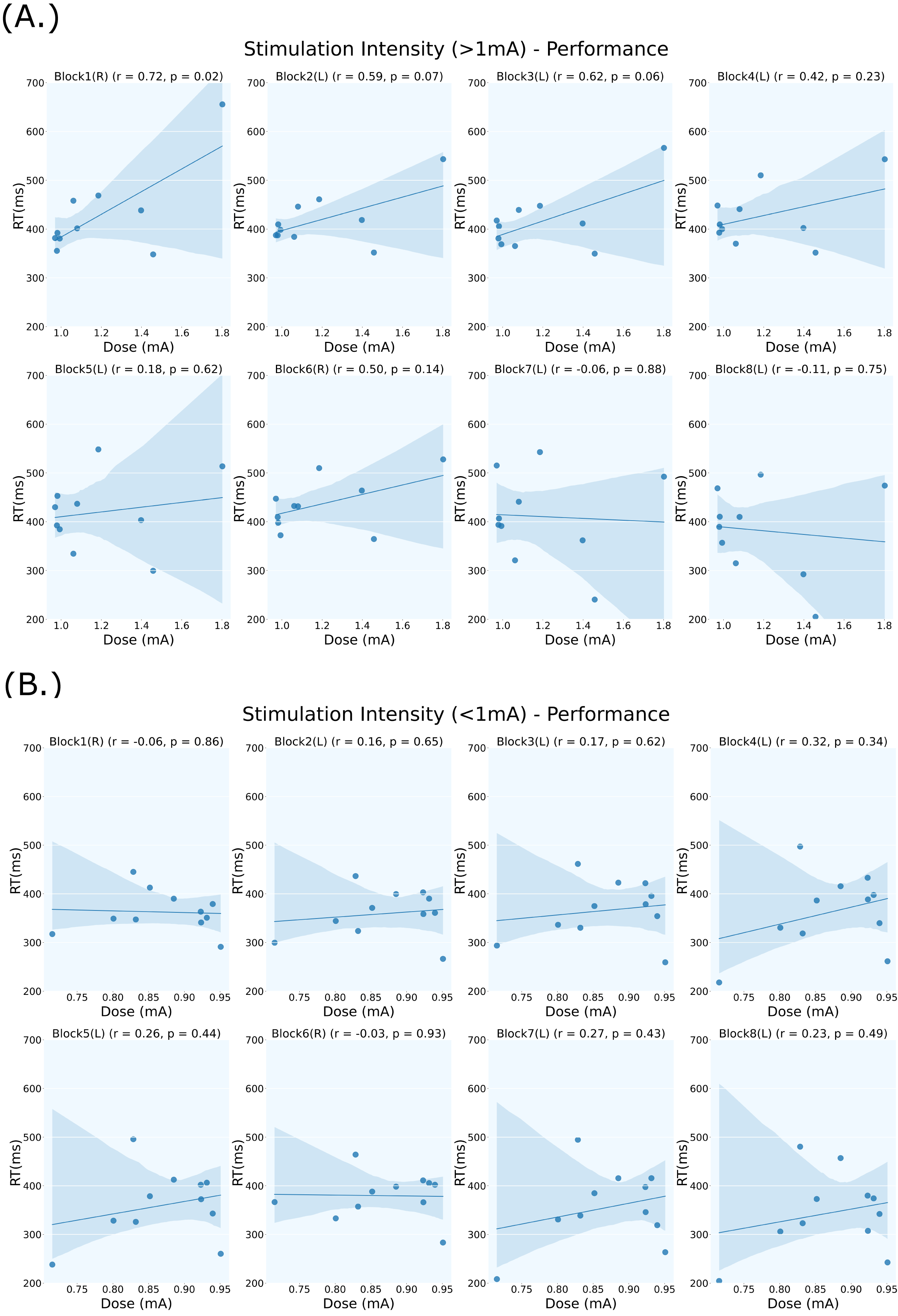

Supplement: Figure 9-3 — A, B, Block-wise correlation between stimulation dose (mA) and the RT (ms) for the cohort with >1 mA (A) and <1 mA (B) stimulation groups. R, Random block; L, learning block. The correlation was nonsignificant for all the blocks. Download Figure 9-3, TIF file. [file enu-eN-NRS-0374-22-s09.tif]

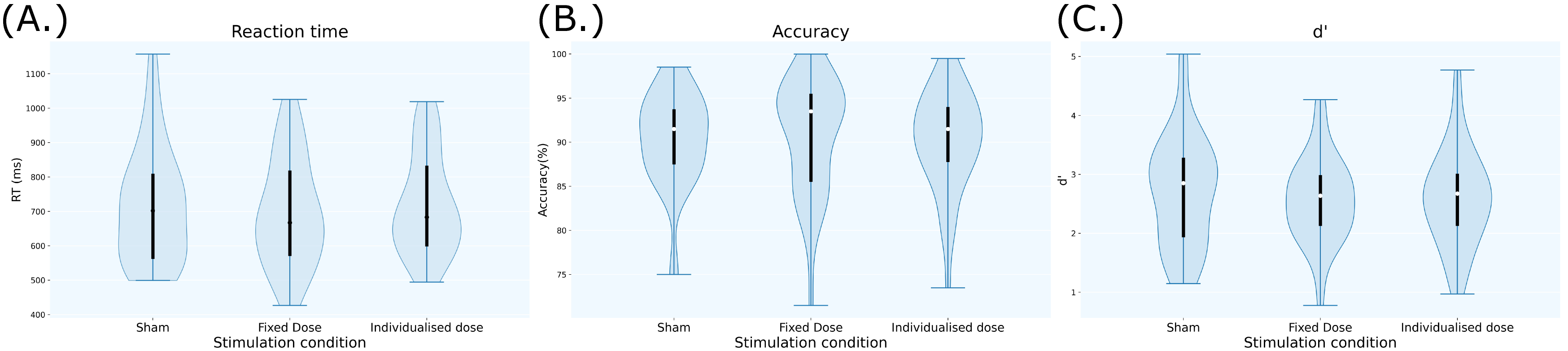

Supplement: Figure 10-1 — Behavioral performance across sham, fixed, and individualized dose tACS conditions. A, RT. B, Accuracy. C, d′. Download Figure 10-1, TIF file. [file enu-eN-NRS-0374-22-s10.tif]
