# Supplementary material for: Behavioral Validation of Individualized Low-Intensity Transcranial Electrical Stimulation (tES) Protocols
Source: eNeuro. 2023 Dec 5;10(12):ENEURO.0374-22.2023. doi: 10.1523/ENEURO.0374-22.2023 (PMC10748339; doi:10.1523/ENEURO.0374-22.2023)
Supplement: Table 2-1. — (a) Post hoc analysis for the main effect of blocks. The Tukey’s HSD was used for comparisons, and the resulting p-values were corrected for multiple comparisons using Bonferroni correction. (b) Post hoc analysis for the main effect of conditions. The Tukey’s HSD was used for comparisons, and the resulting p-values were corrected for multiple comparisons using Bonferroni correction. Download Table 2-1, DOCX file. [file enu-eN-NRS-0374-22-s12.docx]

**Table 2-1(a)**

| **contrast** | **estimate** | **SE** | **df** | **t.ratio** | **p.value** | **d** | **Sig** |
| --- | --- | --- | --- | --- | --- | --- | --- |
| block1 - block2 | 0.0143 | 0.0229 | 480 | 0.6252 | 0.9985 | 0.1114 | - |
| block1 - block3 | 0.0267 | 0.0229 | 480 | 1.1659 | 0.9411 | 0.2077 | - |
| block1 - block4 | 0.0560 | 0.0229 | 480 | 2.4447 | 0.2224 | 0.4356 | - |
| block1 - block5 | 0.0627 | 0.0229 | 480 | 2.7400 | 0.1133 | 0.4882 | - |
| block1 - block6 | -0.0236 | 0.0229 | 480 | -1.0311 | 0.9696 | -0.1837 | - |
| block1 - block7 | 0.0662 | 0.0229 | 480 | 2.8937 | 0.0761 | 0.5156 | - |
| block1 - block8 | 0.0962 | 0.0229 | 480 | 4.2020 | 0.0008 | 0.7487 | *** |
| block2 - block3 | 0.0124 | 0.0229 | 480 | 0.5407 | 0.9994 | 0.0963 | - |
| block2 - block4 | 0.0417 | 0.0229 | 480 | 1.8194 | 0.6071 | 0.3242 | - |
| block2 - block5 | 0.0484 | 0.0229 | 480 | 2.1148 | 0.4068 | 0.3768 | - |
| block2 - block6 | -0.0379 | 0.0229 | 480 | -1.6563 | 0.7154 | -0.2951 | - |
| block2 - block7 | 0.0519 | 0.0229 | 480 | 2.2685 | 0.3133 | 0.4042 | - |
| block2 - block8 | 0.0819 | 0.0229 | 480 | 3.5768 | 0.0091 | 0.6373 | ** |
| block3 - block4 | 0.0293 | 0.0229 | 480 | 1.2788 | 0.9064 | 0.2278 | - |
| block3 - block5 | 0.0360 | 0.0229 | 480 | 1.5741 | 0.7657 | 0.2805 | - |
| block3 - block6 | -0.0503 | 0.0229 | 480 | -2.1970 | 0.3554 | -0.3914 | - |
| block3 - block7 | 0.0396 | 0.0229 | 480 | 1.7278 | 0.6690 | 0.3078 | - |
| block3 - block8 | 0.0695 | 0.0229 | 480 | 3.0361 | 0.0512 | 0.5410 | - |
| block4 - block5 | 0.0068 | 0.0229 | 480 | 0.2953 | 1.0000 | 0.0526 | - |
| block4 - block6 | -0.0796 | 0.0229 | 480 | -3.4758 | 0.0129 | -0.6193 | * |
| block4 - block7 | 0.0103 | 0.0229 | 480 | 0.4490 | 0.9998 | 0.0800 | - |
| block4 - block8 | 0.0402 | 0.0229 | 480 | 1.7573 | 0.6493 | 0.3131 | - |
| block5 - block6 | -0.0863 | 0.0229 | 480 | -3.7711 | 0.0045 | -0.6719 | ** |
| block5 - block7 | 0.0035 | 0.0229 | 480 | 0.1537 | 1.0000 | 0.0274 | - |
| block5 - block8 | 0.0335 | 0.0229 | 480 | 1.4620 | 0.8272 | 0.2605 | - |
| block6 - block7 | 0.0899 | 0.0229 | 480 | 3.9248 | 0.0025 | 0.6993 | ** |
| block6 - block8 | 0.1198 | 0.0229 | 480 | 5.2331 | 0.0000 | 0.9324 | *** |
| block7 - block8 | 0.0300 | 0.0229 | 480 | 1.3083 | 0.8956 | 0.2331 | - |

**p < .05; **p < 0.01; ***p < 0.001*

**Table 2-1(b)**

| **contrast** | **estimate** | **SE** | **df** | **t.ratio** | **p.value** | **d** | **Sig** |
| --- | --- | --- | --- | --- | --- | --- | --- |
| Fixed - Ind | -0.0463 | 0.0140 | 480 | -3.3042 | 0.0029 | -0.3605 | ** |
| Fixed - Sham | -0.0094 | 0.0140 | 480 | -0.6712 | 0.7803 | -0.0732 | - |
| Ind - Sham | 0.0369 | 0.0140 | 480 | 2.6330 | 0.0237 | 0.2873 | * |

**p < .05; **p < 0.01*
