# Supplementary material for: Behavioral Validation of Individualized Low-Intensity Transcranial Electrical Stimulation (tES) Protocols
Source: eNeuro. 2023 Dec 5;10(12):ENEURO.0374-22.2023. doi: 10.1523/ENEURO.0374-22.2023 (PMC10748339; doi:10.1523/ENEURO.0374-22.2023)
Supplement: Table 3-1. — (a) Post hoc analysis for the main effect of blocks. The Tukey’s HSD was used for comparisons, and the resulting p-values were corrected for multiple comparisons using Bonferroni correction. (b) Post hoc analysis for the main effect of conditions. The Tukey’s HSD was used for comparisons, and the resulting p-values were corrected for multiple comparisons using Bonferroni correction. Download Table 3-1, DOCX file. [file enu-eN-NRS-0374-22-s13.docx]

**Table 3-1(a)**

| **contrast** | **estimate** | **SE** | **df** | **t.ratio** | **p.value** | **d** | **Sig** |
| --- | --- | --- | --- | --- | --- | --- | --- |
| block1 - block2 | 0.0180 | 0.0385 | 216 | 0.4673 | 0.9998 | 0.1206 | - |
| block1 - block3 | 0.0335 | 0.0385 | 216 | 0.8692 | 0.9884 | 0.2244 | - |
| block1 - block4 | 0.0356 | 0.0385 | 216 | 0.9236 | 0.9835 | 0.2385 | - |
| block1 - block5 | 0.0593 | 0.0385 | 216 | 1.5382 | 0.7858 | 0.3972 | - |
| block1 - block6 | -0.0124 | 0.0385 | 216 | -0.3209 | 1.0000 | -0.0829 | - |
| block1 - block7 | 0.0734 | 0.0385 | 216 | 1.9050 | 0.5490 | 0.4919 | - |
| block1 - block8 | 0.1212 | 0.0385 | 216 | 3.1472 | 0.0390 | 0.8126 | * |
| block2 - block3 | 0.0155 | 0.0385 | 216 | 0.4019 | 0.9999 | 0.1038 | - |
| block2 - block4 | 0.0176 | 0.0385 | 216 | 0.4564 | 0.9998 | 0.1178 | - |
| block2 - block5 | 0.0413 | 0.0385 | 216 | 1.0710 | 0.9621 | 0.2765 | - |
| block2 - block6 | -0.0304 | 0.0385 | 216 | -0.7882 | 0.9936 | -0.2035 | - |
| block2 - block7 | 0.0554 | 0.0385 | 216 | 1.4377 | 0.8387 | 0.3712 | - |
| block2 - block8 | 0.1032 | 0.0385 | 216 | 2.6799 | 0.1345 | 0.6919 | - |
| block3 - block4 | 0.0021 | 0.0385 | 216 | 0.0544 | 1.0000 | 0.0141 | - |
| block3 - block5 | 0.0258 | 0.0385 | 216 | 0.6690 | 0.9977 | 0.1727 | - |
| block3 - block6 | -0.0459 | 0.0385 | 216 | -1.1901 | 0.9341 | -0.3073 | - |
| block3 - block7 | 0.0399 | 0.0385 | 216 | 1.0358 | 0.9685 | 0.2674 | - |
| block3 - block8 | 0.0878 | 0.0385 | 216 | 2.2780 | 0.3106 | 0.5882 | - |
| block4 - block5 | 0.0237 | 0.0385 | 216 | 0.6146 | 0.9987 | 0.1587 | - |
| block4 - block6 | -0.0479 | 0.0385 | 216 | -1.2446 | 0.9175 | -0.3213 | - |
| block4 - block7 | 0.0378 | 0.0385 | 216 | 0.9813 | 0.9767 | 0.2534 | - |
| block4 - block8 | 0.0857 | 0.0385 | 216 | 2.2235 | 0.3419 | 0.5741 | - |
| block5 - block6 | -0.0716 | 0.0385 | 216 | -1.8592 | 0.5804 | -0.4800 | - |
| block5 - block7 | 0.0141 | 0.0385 | 216 | 0.3667 | 1.0000 | 0.0947 | - |
| block5 - block8 | 0.0620 | 0.0385 | 216 | 1.6089 | 0.7445 | 0.4154 | - |
| block6 - block7 | 0.0858 | 0.0385 | 216 | 2.2259 | 0.3405 | 0.5747 | - |
| block6 - block8 | 0.1336 | 0.0385 | 216 | 3.4681 | 0.0144 | 0.8955 | * |
| block7 - block8 | 0.0479 | 0.0385 | 216 | 1.2422 | 0.9183 | 0.3207 | - |

**p < .05*

**Table 3-1(b)**

| **contrast** | **estimate** | **SE** | **df** | **t.ratio** | **p.value** | **d** | **Sig** |
| --- | --- | --- | --- | --- | --- | --- | --- |
| Fixed - Ind | -0.0567 | 0.0236 | 216 | -2.4038 | 0.0448 | -0.3801 | * |
| Fixed - Sham | -0.0289 | 0.0236 | 216 | -1.2269 | 0.4386 | -0.1940 | - |
| Ind - Sham | 0.0278 | 0.0236 | 216 | 1.1769 | 0.4682 | 0.1861 | - |

**p < 0.05*
