# Supplementary material for: Behavioral Validation of Individualized Low-Intensity Transcranial Electrical Stimulation (tES) Protocols
Source: eNeuro. 2023 Dec 5;10(12):ENEURO.0374-22.2023. doi: 10.1523/ENEURO.0374-22.2023 (PMC10748339; doi:10.1523/ENEURO.0374-22.2023)
Supplement: Table 4-1. — (a) Post hoc analysis for the main effect of blocks. The Tukey’s HSD was used for comparisons, and the resulting p-values were corrected for multiple comparisons using Bonferroni correction. (b) Post hoc analysis for the main effect of conditions. The Tukey’s HSD was used for comparisons, and the resulting p-values were corrected for multiple comparisons using Bonferroni correction. Download Table 4-1, DOCX file. [file enu-eN-NRS-0374-22-s14.docx]

**Table 4-1(a)**

| **contrast** | **estimate** | **SE** | **df** | **t.ratio** | **p.value** | **d** | **Sig** |
| --- | --- | --- | --- | --- | --- | --- | --- |
| block1 - block2 | 0.0110 | 0.0274 | 240 | 0.3993 | 0.9999 | 0.0983 | - |
| block1 - block3 | 0.0205 | 0.0274 | 240 | 0.7473 | 0.9954 | 0.1840 | - |
| block1 - block4 | 0.0745 | 0.0274 | 240 | 2.7141 | 0.1234 | 0.6682 | - |
| block1 - block5 | 0.0659 | 0.0274 | 240 | 2.4001 | 0.2461 | 0.5909 | - |
| block1 - block6 | -0.0338 | 0.0274 | 240 | -1.2324 | 0.9215 | -0.3034 | - |
| block1 - block7 | 0.0597 | 0.0274 | 240 | 2.1769 | 0.3695 | 0.5359 | - |
| block1 - block8 | 0.0734 | 0.0274 | 240 | 2.6751 | 0.1354 | 0.6586 | - |
| block2 - block3 | 0.0096 | 0.0274 | 240 | 0.3480 | 1.0000 | 0.0857 | - |
| block2 - block4 | 0.0635 | 0.0274 | 240 | 2.3148 | 0.2899 | 0.5699 | - |
| block2 - block5 | 0.0549 | 0.0274 | 240 | 2.0008 | 0.4835 | 0.4926 | - |
| block2 - block6 | -0.0448 | 0.0274 | 240 | -1.6317 | 0.7307 | -0.4017 | - |
| block2 - block7 | 0.0488 | 0.0274 | 240 | 1.7776 | 0.6359 | 0.4376 | - |
| block2 - block8 | 0.0625 | 0.0274 | 240 | 2.2758 | 0.3114 | 0.5603 | - |
| block3 - block4 | 0.0540 | 0.0274 | 240 | 1.9668 | 0.5066 | 0.4842 | - |
| block3 - block5 | 0.0454 | 0.0274 | 240 | 1.6527 | 0.7175 | 0.4069 | - |
| block3 - block6 | -0.0543 | 0.0274 | 240 | -1.9797 | 0.4978 | -0.4874 | - |
| block3 - block7 | 0.0392 | 0.0274 | 240 | 1.4295 | 0.8428 | 0.3519 | - |
| block3 - block8 | 0.0529 | 0.0274 | 240 | 1.9277 | 0.5332 | 0.4746 | - |
| block4 - block5 | -0.0086 | 0.0274 | 240 | -0.3140 | 1.0000 | -0.0773 | - |
| block4 - block6 | -0.1083 | 0.0274 | 240 | -3.9465 | 0.0026 | -0.9716 | ** |
| block4 - block7 | -0.0147 | 0.0274 | 240 | -0.5372 | 0.9994 | -0.1323 | - |
| block4 - block8 | -0.0011 | 0.0274 | 240 | -0.0390 | 1.0000 | -0.0096 | - |
| block5 - block6 | -0.0997 | 0.0274 | 240 | -3.6325 | 0.0081 | -0.8943 | ** |
| block5 - block7 | -0.0061 | 0.0274 | 240 | -0.2232 | 1.0000 | -0.0549 | - |
| block5 - block8 | 0.0075 | 0.0274 | 240 | 0.2750 | 1.0000 | 0.0677 | - |
| block6 - block7 | 0.0936 | 0.0274 | 240 | 3.4093 | 0.0172 | 0.8393 | * |
| block6 - block8 | 0.1072 | 0.0274 | 240 | 3.9075 | 0.0030 | 0.9619 | ** |
| block7 - block8 | 0.0137 | 0.0274 | 240 | 0.4982 | 0.9997 | 0.1226 | - |

**p < .05; **p < 0.01*

**Table 4-1(b)**

| **contrast** | **estimate** | **SE** | **df** | **t.ratio** | **p.value** | **d** | **Sig** |
| --- | --- | --- | --- | --- | --- | --- | --- |
| Fixed - Ind | -0.0369 | 0.0168 | 240 | -2.1940 | 0.0742 | -0.3308 | - |
| Fixed - Sham | 0.0083 | 0.0168 | 240 | 0.4968 | 0.8729 | 0.0749 | - |
| Ind - Sham | 0.0452 | 0.0168 | 240 | 2.6908 | 0.0208 | 0.4056 | * |

**p < .05*
