# Supplementary material for: Behavioral Validation of Individualized Low-Intensity Transcranial Electrical Stimulation (tES) Protocols
Source: eNeuro. 2023 Dec 5;10(12):ENEURO.0374-22.2023. doi: 10.1523/ENEURO.0374-22.2023 (PMC10748339; doi:10.1523/ENEURO.0374-22.2023)
Supplement: Table 5-1. — (a) Post hoc analysis for the main effect of blocks. The Tukey’s HSD was used for comparisons, and the resulting p-values were corrected for multiple comparisons using Bonferroni correction. (b) Post hoc analysis for the main effect of sessions. The Tukey’s HSD was used for comparisons, and the resulting p-values were corrected for multiple comparisons using Bonferroni correction. Download Table 5-1, DOCX file. [file enu-eN-NRS-0374-22-s15.docx]

**Table 5-1(a)**

| **contrast** | **estimate** | **SE** | **df** | **t.ratio** | **p.value** | **d** | **Sig** |
| --- | --- | --- | --- | --- | --- | --- | --- |
| Block1 - Block2 | 0.0143 | 0.0227 | 480 | 0.6298 | 0.9985 | 0.1122 | - |
| Block1 - Block3 | 0.0267 | 0.0227 | 480 | 1.1744 | 0.9389 | 0.2092 | - |
| Block1 - Block4 | 0.0560 | 0.0227 | 480 | 2.4624 | 0.2143 | 0.4387 | - |
| Block1 - Block5 | 0.0627 | 0.0227 | 480 | 2.7599 | 0.1078 | 0.4917 | - |
| Block1 - Block6 | -0.0236 | 0.0227 | 480 | -1.0386 | 0.9683 | -0.1851 | - |
| Block1 - Block7 | 0.0662 | 0.0227 | 480 | 2.9147 | 0.0719 | 0.5193 | - |
| Block1 - Block8 | 0.0962 | 0.0227 | 480 | 4.2325 | 0.0007 | 0.7541 | *** |
| Block2 - Block3 | 0.0124 | 0.0227 | 480 | 0.5446 | 0.9994 | 0.0970 | - |
| Block2 - Block4 | 0.0417 | 0.0227 | 480 | 1.8327 | 0.5980 | 0.3265 | - |
| Block2 - Block5 | 0.0484 | 0.0227 | 480 | 2.1301 | 0.3970 | 0.3795 | - |
| Block2 - Block6 | -0.0379 | 0.0227 | 480 | -1.6683 | 0.7078 | -0.2973 | - |
| Block2 - Block7 | 0.0519 | 0.0227 | 480 | 2.2849 | 0.3040 | 0.4071 | - |
| Block2 - Block8 | 0.0819 | 0.0227 | 480 | 3.6028 | 0.0083 | 0.6419 | ** |
| Block3 - Block4 | 0.0293 | 0.0227 | 480 | 1.2881 | 0.9031 | 0.2295 | - |
| Block3 - Block5 | 0.0360 | 0.0227 | 480 | 1.5855 | 0.7589 | 0.2825 | - |
| Block3 - Block6 | -0.0503 | 0.0227 | 480 | -2.2130 | 0.3457 | -0.3943 | - |
| Block3 - Block7 | 0.0396 | 0.0227 | 480 | 1.7403 | 0.6607 | 0.3101 | - |
| Block3 - Block8 | 0.0695 | 0.0227 | 480 | 3.0582 | 0.0480 | 0.5449 | * |
| Block4 - Block5 | 0.0068 | 0.0227 | 480 | 0.2975 | 1.0000 | 0.0530 | - |
| Block4 - Block6 | -0.0796 | 0.0227 | 480 | -3.5010 | 0.0118 | -0.6238 | * |
| Block4 - Block7 | 0.0103 | 0.0227 | 480 | 0.4523 | 0.9998 | 0.0806 | - |
| Block4 - Block8 | 0.0402 | 0.0227 | 480 | 1.7701 | 0.6407 | 0.3154 | - |
| Block5 - Block6 | -0.0863 | 0.0227 | 480 | -3.7985 | 0.0041 | -0.6768 | ** |
| Block5 - Block7 | 0.0035 | 0.0227 | 480 | 0.1548 | 1.0000 | 0.0276 | - |
| Block5 - Block8 | 0.0335 | 0.0227 | 480 | 1.4726 | 0.8217 | 0.2624 | - |
| Block6 - Block7 | 0.0899 | 0.0227 | 480 | 3.9533 | 0.0022 | 0.7044 | ** |
| Block6 - Block8 | 0.1198 | 0.0227 | 480 | 5.2711 | 0.0000 | 0.9392 | *** |
| Block7 - Block8 | 0.0300 | 0.0227 | 480 | 1.3178 | 0.8919 | 0.2348 | - |

**p < .05; **p < 0.01; ***p < 0.001*

**Table 5-1(b)**

| **contrast** | **estimate** | **SE** | **df** | **t.ratio** | **p.value** | **d** | **Sig** |
| --- | --- | --- | --- | --- | --- | --- | --- |
| Session 1 - Session 2 | -0.0541 | 0.0139 | 480 | -3.8853 | 0.0003 | -0.4239 | *** |
| Session 1 - Session 3 | -0.0528 | 0.0139 | 480 | -3.7902 | 0.0005 | -0.4136 | *** |
| Session 2 - Session 3 | 0.0013 | 0.0139 | 480 | 0.0951 | 0.9950 | 0.0104 | - |

****p < 0.001*
